# Oral Supplements of Combined *Lactobacillus plantarum* and *Asparagus officinalis* Modulate Gut Microbiota and Alleviate High-Fat Diet–Induced Cognitive Deficits and Neurodegeneration in Rats

**DOI:** 10.1007/s12602-024-10429-7

**Published:** 2025-01-07

**Authors:** Nancy N. Shahin, Omar A. Ahmed‐Farid, Ebtehag A. E. Sakr, Enas A. Kamel, Maha M. Mohamed

**Affiliations:** 1 Department of Biochemistry, Faculty of Pharmacy, Cairo University, Cairo, 11562 Egypt; 2 Department of Physiology, Egyptian Drug Authority, Giza, 12553 Egypt; 3 Botany Department, Faculty of Women for Arts, Science and Education, Ain Shams University, Cairo, Egypt; 4 Biochemistry and Nutrition Department, Faculty of Women for Arts Science and Education, Ain Shams University, Cairo, Egypt; 5 Home Economic Department, Faculty of Women for Arts Science and Education, Ain Shams University, Cairo, Egypt

**Keywords:** *Asparagus officinalis*, *Lactobacillus plantarum* 20174, Neurodegeneration, High-fat diet, Microbiota

## Abstract

High-fat diet (HFD) consumption disrupts the gut microbiome, instigating metabolic disturbance, brain pathology, and cognitive decline via the gut-brain axis. Probiotic and prebiotic supplementation have been found to improve gut microbiome health, suggesting they could be effective in managing neurodegenerative disorders. This study explored the potential benefits of the probiotic strain *Lactobacillus plantarum* 20174 (*L. plantarum*), prebiotic *Asparagus officinalis* (*A. officinalis*) extract, or their synbiotic combination against HFD-induced cognitive dysfunction and neurodegeneration in rats. Male Sprague–Dawley rats were fed either a normal diet or an HFD for 24 weeks. Starting from week 13, rats on either diet were divided into vehicle-, prebiotic-, probiotic-, and synbiotic-treated subgroups. Rats received their assigned intervention for 12 more weeks. Prebiotic, probiotic, or synbiotic treatment reverted HFD-instigated alterations in hippocampal amyloid beta, p-tau, α-synuclein, and BDNF levels, leading to restored cognitive function. The tested therapies also improved the HFD-disrupted lipid profile. Interestingly, probiotic and synbiotic therapies attenuated oxidative stress and inflammation, reinstated neurotransmitter balance, and mitigated the energy deficit in HFD-fed rats. Furthermore, *L. plantarum* and *Asparagus* administration modulated gut microbiota composition by raising *Lactobacillus* species and reducing Coliform and *Staphylococci* bacteria as well as fungi populations. These findings suggest that the oral consumption of *A. officinalis* prebiotics and/or *L. plantarum* probiotics alleviates HFD-induced cognitive deficit and neurodegeneration through modulation of the gut-brain axis with superior restorative effects being achieved by synbiotic treatment.

**Graphical Abstract:**

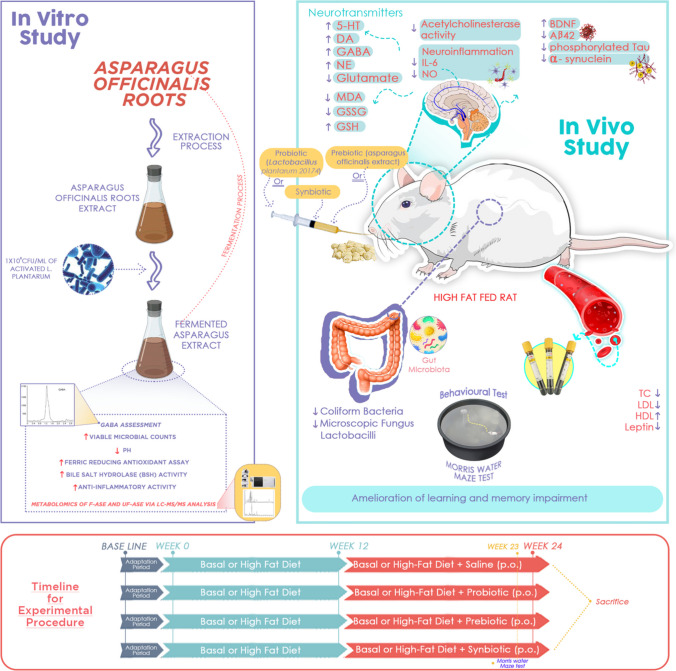

## Introduction

Neurodegenerative disorders and associated cognitive decline are significant contributing risk factors to morbidity and the declining quality of life for millions worldwide [1]. Accumulating evidence has demonstrated that obesity and high fat intake are elements of risk for neurodegenerative disorders and cognitive deterioration [2]. An epidemiological study revealed that obese people who consume high-fat diets (HFDs) demonstrate impaired mental function [3]. Also, animal studies supported the notion that obesity induced by the consumption of HFD caused hippocampal synaptic malfunction manifested by the loss of dendritic spine and impairment of long-term potentiation (LTP), contributing to cognitive deterioration [4, 5]. Growing evidence has revealed that altering the gut-brain axis is implicated in obesity-induced cognitive decline [4, 6]. For instance, HFD-induced gut microbiome alterations impaired cognitive function in mice [6]. Moreover, it has been demonstrated that transplanting an obese-type microbiota might disintegrate the intestinal barrier and cause cognitive impairment in mice [7]. Hence, employing probiotics or prebiotics to modulate gut microbiota has emerged as a possible therapy for HFD intake-associated cognitive impairment.

Probiotics are living microorganisms that, when supplied in sufficient amounts, confer health-promoting benefits in the host by maintaining the integrity of gut microflora, lowering bacterial translocation, and averting infection. *Lactobacillus* is mainly utilized as a probiotic; specifically, the plantarum strain is a common bacteria found in meat, dairy products, fruits, and vegetables [8]. It has been reported that feeding *L. plantarum* modulated the immune state of animals, resulting in boosting host immunity through the expression of immunological factors in immune organs and altering the activity of specific immune cells and the levels of antimicrobial substances and immunoglobulin in blood [9]. Furthermore, *Lactobacillus* has been shown to modify gut microbiota and exert antioxidant effects by adjusting the redox status through scavenging free radicals, chelating metal ions, and regulating antioxidant enzymes in various host tissues [10, 11]. Moreover, Lactobacilli have been associated with reduced inflammatory cytokines [12, 13] and enhanced brain-derived neurotrophic factor (BDNF) levels in rats’ hippocampi [14].

Prebiotics are non-digestible food components that selectively boost the activity and growth of beneficial bacterial species already established in the colon, thereby enhancing the host’s health [15]. Asparagus roots and rhizome are by-products of asparagus cultivation, that could serve as alternative sources of inulin-type fructans [16]. Natural inulin-type fructans, like dietary fibers, are not digested by human digestive enzymes. Instead, they are metabolized by the intestinal microbiota and possess prebiotic properties that promote the flourishing of probiotics and the production of beneficial short-chain fatty acids (SCFAs) [17]. A natural fructan obtained from the roots of *Asparagus cochinchinensis* was fermented in vitro by human fecal microbiota. The researchers in this study observed a drop in the pH of the culture medium, coinciding with an increase in the content of SCFAs, particularly acetic, propionic, n-valeric acids, and i-valeric. They also observed significant changes in the microbiota composition after a 24-h incubation period: the genus Haemophilus, associated with respiratory and neurological disorders, decreased, while the beneficial genera Prevotella, Megamonas, and Bifidobacterium increased [18]. These results indicated a health-promoting effect associated with the consumption of Asparagus fructan.

When probiotics are combined with prebiotic formulations, the resulting functional products constitute synbiotics. The synbiotic concept was recently revised to be a mixture of living microorganisms and the substrate that the host microorganisms specifically utilize to promote the host’s health [19]. Such preparations could be designed in complementarity that targets the host microbiota or in synergism for which the co-administrated probiotics selectively utilize the prebiotic to achieve one or more health benefits. It has been shown that certain probiotic strains exhibit discernible variations in their capacities to ferment various oligosaccharides to promote their growth [20], implying that using probiotics and prebiotics in random combinations may not always produce the intended outcome. Wang et al. assessed the possible synbiotic effects of treating weaning piglets with *L. plantarum* ZLP001 combined with selected fructooligosaccharides. They reported that fructooligosaccharide could be well utilized by *Lactobacillus plantarum* ZLP001 and can be combined with it as a possible synbiotic that shows synergistic beneficial effects in piglets [21].

To our knowledge, the effects of the probiotic *L. plantarum*, the prebiotic Asparagus, or their synbiotic combination on the modulation of cognitive function by altering intestinal microbiota in high-fat-fed rats are yet to be studied. Therefore, the present study aimed to investigate whether the consumption of *L. plantarum* DMS 20174, *Asparagus officinalis* extract, or their combination could alleviate HFD-induced neurodegeneration and cognitive decline in rats by manipulating gut microbiota dysbiosis. This was accomplished by evaluating the memory and spatial learning of rats, colon microbial count, serum lipid profile, striatal and hippocampal acetylcholinesterase, neurotransmitters, inflammatory, oxidative stress, and energy markers, in addition to the hippocampal levels of the neurotrophin, BDNF, and the biomarkers of neurodegeneration, α-synuclein, phosphorylated tau and beta-amyloid 1–42. Additionally, we assessed the in vitro viable microbial counts, pH, GABA, antioxidant and anti-inflammatory activities, and the metabolomics of ASE fermented by *L. plantarum*. The bile salt hydrolase activity of *L. plantarum* grown on ASE was also determined.

## Materials and Methods

### Collection of *A. officinalis* Tubers

Fresh *A. officinalis* tubers were collected from the Egyptian Plant Nursery during harvest. The harvested tubers were cleaned, washed with tap water to remove the remaining soil, manually peeled, then cut into little pieces, and the whole pulp was homogenized using a food processor. The homogenized plant was filtered, and the filtrate was dehydrated in a cabinet drier for 24 h. The dried samples (*A. officinalis* extract, ASE) were ground in an electric mill until they could pass through a 60 mesh filter [22].

### Bacterial Strain and Its Preparation

Pure culture of *L. plantarum* DMS 20174 was purchased from the Faculty of Agriculture, Ain-Shams University, Egypt. Before use, the strain was subcultured three times in sterile De Man, Rogosa, and Sharpe (MRS) broth using a 1% inoculum and a 24-h incubation period at 37 °C*. L. plantarum* was prepared in accordance with a previously published investigation (12 × 10^8^ colony-forming unit (CFU)/mL; 10 mL /kg body weight) for the in vivo experiment [23].

### The *In Vitro* Study

#### The Fermentation Experiment

Fermentation media were prepared by adding 5% ASE and 2% peptone [24]. The pH of the medium was manipulated to 6 ± 0.5 using 1N NaOH. They were well mixed, autoclaved at 121 °C for 15 min, and cooled to 30 °C. Then, the sterilized ASE and MRS media were inoculated with a probiotic culture of *L. plantarum* (1 × 10^8^ CFU/mL). Thereafter, the inoculated media were fermented in a 37° C incubator for 72 h. Then, the fermented media were subjected to microbiological pH, GABA, antioxidant, bile salt hydrolase, and anti-inflammatory activities at different intervals (0, 24, 48, 72 h).

#### Determination of Viable Microbial Counts, pH, and GABA

At 0, 24, 48, and 72 h of fermentation, the viable counts, pH, and GABA levels of *L. plantarum*–fermented *A. officinalis* extract (F-ASE) and MRS (F-MRS) were measured. For viable counts, 1 mL of each of the F-ASE and F-MRS was added to 9 mL of sterile saline and vortexed. On a tenfold gradient, the various samples were diluted, and the dilutions were then transferred to MRS-agar plates. Bacterial strains (log CFU/mL) were counted after 48 h of 37 °C incubation. Each sample’s pH was determined by centrifuging 5 mL of it using a pH meter (Beckman, USA).

Cultures obtained from fermented *A. officinalis* extract (F-ASE) and fermented MRS (F-MRS) at 24, 48, and 72 h were used to quantitatively determine GABA concentration. Centrifugation was used to separate the culture broth from the cells (6000 rpm at 4 °C for 10 min), and a 0.45-m membrane filter (CHMLAP Group) was applied to filter the supernatant. After that, the supernatant was diluted 1:1. Pre-column derivatization with the o-phthaldialdehyde reagent was used to measure the GABA concentrations in the diluted supernatant [25]. Chromatographic analysis was completed using an Agilent HPLC system (HP 1100 series), which was outfitted with a quaternary pump, auto-sampler, online degasser, and diode array detector. The system was controlled by Chemstation software from Hewlett Packard, Wald Bronn, Germany. The UV detector (at 254 nm) operated for 0–28 min at a column temperature of 30 °C. Tetrahydrofuran (10 mL), methanol (490 mL), and sodium acetate (0.05 M, 500 mL) were used as the mobile phase. After that, a 0.2-m membrane filter was used to filter the mixed solution, and sonication was used for degassing it [26]. The flow rate and GABA standard concentration were 1 mL min^−1^ and 1 µg/mL, respectively.

#### Antioxidant Assay

The antioxidant activity was determined as previously described [27]. After 72 h of incubation, 100 μL of the cell-free supernatant (CFS) from variously diluted F-ASE and F-MRS medium was combined with 1% K_3_FeCN_6_ for 20 min at 50 °C. Trichloroacetic acid (10%) was subsequently added to the mixture after it had cooled. The upper layer was extracted from the centrifuged content and combined with a 0.1% FeCl_3_ solution. The reducing power was then expressed as the average reading for absorbance at 700 nm. Ascorbic acid was used as a reference (positive control).

#### Determination of Bile Salt Hydrolase (BSH) Activity

The cultures of *L. plantarum* grown on ASE and MRS medium were utilized to assess BSH activity after 72 h of incubation. Agar (1% w/v) was added to MRS broth together with bile salts (0.3% w/v) and calcium chloride (0.375 g/L) to create soft MRS agar. The culture cells (50, 100, 150, and 200 µL) were placed into holes. The plates were placed in an incubator for 72 h at 37 °C after spending 10 min in the laminar flow. The negative control was MRS agar without bile salts. The BSH activity was detected by the formation of translucent haloes surrounding holes [28].

#### Determination of Anti-inflammatory Activity

According to the previously outlined procedure [29], the protocols for studying the anti-inflammatory activity were carried out. Cell viability was evaluated using the 3-(4,5-dimethylthiazol-2-yl)−2,5 diphenyltetrazolium bromide (MTT) assay (5 mg/mL). In 96-well plates, RAW264.7 cells were plated at a density of 0.5 × 10^6^ cells/mL for 24 h. The cells were subsequently induced with 100 ng/mL of lipopolysaccharide (LPS) for 24 h after receiving a pretreatment (30 min at 37 °C) with sterile F-ASE (10%, 5%, 2.5%, 1.25%, and 0.62% v/v). The Griess reaction was used to calculate the NO level. Griess reagent was briefly mixed with cell culture supernatant in equal amounts for 10 min, and then the absorbance (520 nm) was calculated using a microplate reader. Nω-Nitro-L-arginine methyl ester hydrochloride (L-NAME, 1 mM) was utilized as an anti-inflammatory agent (positive control). Compared to the LPS-induced inflammation group, the NO inhibition % of test samples was evaluated and standardized to cell viability as assessed by the Alamar Blue™ reduction assay [30].

#### Metabolomics of F-ASE and UF-ASE

Using liquid chromatography/mass spectrometry tandem device (Nexera with LCMS-8045, Shimadzu Corporation, Kyoto, Japan), the metabolic profiles of the F-ASE after 72 h of incubation and the unfermented asparagus extract (UF-ASE) were determined. The C_18_ column (Shimpack-RP-C18 UPLC 2 150 mm—2.7 m particle size) was used for the separation. A several-step linear gradient composed of eluent B (acetonitrile + 0.1% formic acid) and the mobile phase A (water + 0.1% formic acid) was used. A 5 µL aliquot of each extract was injected with 0.2 mL/min flow rate. While using electrospray ionization (ESI) and LC–MS/MS, positive and negative modes were in operation. Lab Solutions software was used to gather and process the LC–MS/MS data (Shimadzu, Kyoto, Japan).

### The *In Vivo* Study

#### Animals and Experimental Design

Forty-eight male Sprague–Dawley rats, weighing 140 to 150 g, were procured from Serum and Vaccine Farm, Helwan, Cairo, Egypt. The animals were kept separately within metal cages at a regulated ambient temperature (23 to 25 °C) and a set artificial light/dark cycle of 12:12 h. Water and basal diet were supplied ad libitum for a week as an adaptation time according to AIN-93 recommendations [31]. All animal experiments in the current study were authorized by the Research Ethics Committee of the Faculty of Pharmacy, Cairo University (Permit Number BC 3356).

**Fig. 1:**
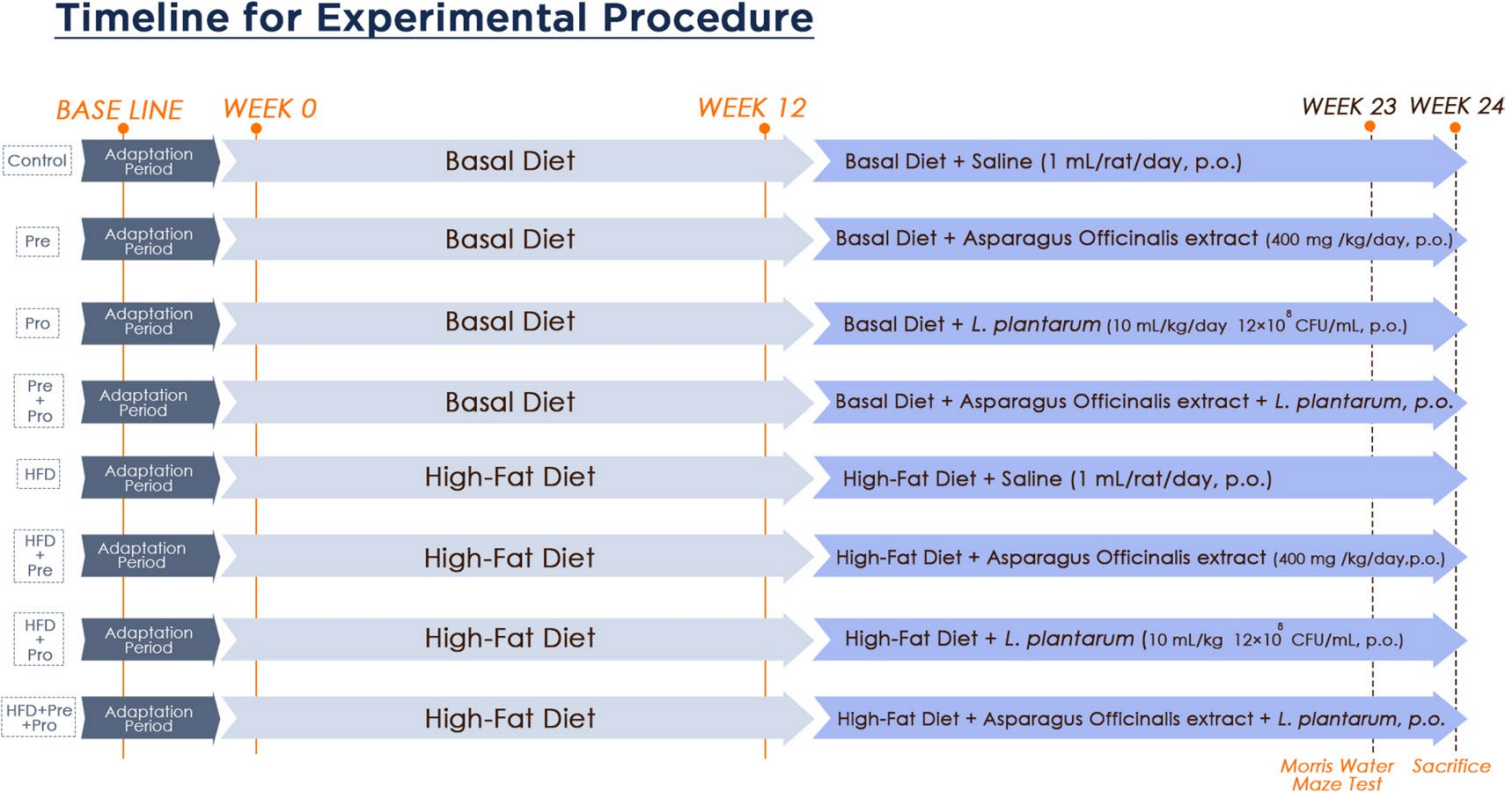
In vivo study design

Following a week of adaptation, rats were randomly assigned into two groups (24 rats in each group). They were supplied with either a basal diet (BD) or a high-fat diet (HFD) containing 15% tallow, 7.5% corn oil, and 1% cholesterol for 24 weeks [32]. Starting from week 13, each of the BD- and the HFD-fed groups was sub-grouped into four groups (*n* = 6) and treated by oral gavage daily with 1 mL saline (control), asparagus extract (400 mg/kg body weight) [33], *Lactobacillus plantarum* (10 mL/kg body weight; 12 × 10^8^ CFU/mL) [23], or a mixture of both, up to the end of the experiment, as illustrated in Fig. 1.

#### Behavioral Tests

Memory and spatial learning of rats were evaluated utilizing the Morris Water Maze procedure [34, 35]. Throughout the last 4 days of the experiment, there were two daily trials, lasting 60 s each. A 120-cm-diameter, 0.5-m-high circular black pool was employed. An escape platform measuring 14 cm by 14 cm was created for the maze. The platform was covered with 1 cm of water. The first rat was carefully put in one of the quadrants, and a timer was used to record the time it took to locate the platform during latency. After 60 s, if the rat could not locate the platform, it was removed from the water and placed on it. The animal was taken out of the maze after being permitted to remain on the platform for 10 s. Then, the rat was dried and given 10 min before being put in the pool for the second trial. The trials were conducted again until the rats located the platform. For the other rats, the exact protocol was used. The learning curve was plotted for every rat. The trial number was shown on the X-axis, while the time to find the platform was on the Y-axis. On the fourth day, a probe trial was conducted in which the rats were permitted to swim without a platform in the pool for 60 s.

#### Sampling

At the end of the investigational period, and after behavioral testing, blood samples were drawn from the retro-orbital plexus, under thiopental anesthesia (50 mg/kg, i.p.) [36], in sanitized gel tubes, and were thereafter processed for the separation of serum that was kept at − 80 °C for subsequent lipid profile and leptin analyses. Afterwards, rats were euthanized by decapitation, then liver, spleen, kidney, and colon samples were rapidly excised, rinsed with ice-cold 0.9% sodium chloride solution, blotted using filter paper, and weighed for the subsequent microbiological analyses. The brains were exposed and the striata and hippocampi were promptly dissected and stored at − 80 °C for later biochemical analyses.

#### Microbiological Analyses

After being dissected, the tissues from the colon were collected into sterile containers and later combined for microbial analysis. One gram of the colon’s feces was put into a test tube with 9 mL of sterile peptone water and homogenized by vortexing. To determine the total aerobic bacterial counts, a tenfold serial dilution was carried out, and also the relevant dilutions were pour plated on nutrient agar. The number of Lactobacilli was counted using Man Rogosa (MRS) agar. On MacConkey agar, Enterobacteriaceae were counted. The number of Staphylococci was counted on mannitol salt agar. Plates containing Staphylococci, Lactobacilli, Enterobacteriaceae, and total aerobes were incubated at 37 °C for 48 h. Sabouraud agar was used to count microscopic fungus that had been cultured at 30 °C for 5 days [37].

The kidneys, spleen, and liver were all removed, blotted on filter paper, and stored in separate sterile tubes. To determine translocation, the presence of Lactobacilli was examined in all livers, spleens, and kidneys [37].

#### Biochemical Analysis

##### Determination of Serum Lipid Profile and Leptin Level

The enzymatic colorimetric standard kits were utilized to evaluate the concentrations of total cholesterol (TC), high-density lipoprotein cholesterol (HDL-C), and triacylglycerol (TG) in serum, following the colorimetric methods according to the manufacturer’s instructions. The serum LDL-C value was determined using the formula [LDL-C = TC-(HDL-C + TG/5)] [38]. Meanwhile, the serum level of leptin was evaluated using a rat-specific enzyme-linked immunosorbent assay (ELISA) kit supplied by Cusabio (Catalog No. CSB-E07433r, Houston, TX, USA), employing the sandwich technique. The assay procedure was carried out in accordance with the manufacturer’s recommendations.

##### Determination of Hippocampal Acetylcholinesterase (AChE) Activity

Acetylcholinesterase activity determination was conducted according to the method described by Gorun et al. [39]. Briefly, 0.1 mL of 5,5-dithiobis (2-nitrobenzoate) and 2.7 mL of phosphate buffer were added to the tissue homogenate. Then, 0.1 mL of freshly prepared acetylcholine iodide with pH 8 was added. The absorbance of the produced color was measured immediately at 412 nm.

##### HPLC Determination of Striatal and Hippocampal Levels of Neurotransmitters, Nitric Oxide, Oxidative Stress, and Energy Markers

The striatal and hippocampal tissues were weighed and homogenized in 75% methanol of HPLC grade (10% w/v). After homogenizing the tissues, they were centrifuged, and the supernatants were utilized for HPLC–UV investigation. For measurement of striatal and hippocampal levels of reduced/oxidized glutathione (GSH/GSSG), malondialdehyde (MDA), nitrite/nitrate (NO), 8**-**hydroxy**-**2′**-**deoxyguanosine (8-OHdG), monoamine neurotransmitters and their metabolites, gamma-aminobutyric acid (GABA), and glutamic acid (Glu), in addition to energy makers, HPLC device (Agilent HP 1200 series, USA) was used. The apparatus included a column oven, quaternary pump, Rheodine injector 20 µL loop, and UV variable wavelength detector. The chromatograms and reports were obtained using the Chemstation software (Agilent, USA).

For GSH and GSSG measurements, the analytical μBondapak column (15 cm × 3.9 mm) was used, and the mobile phase was 25 mmol sodium phosphate buffer (pH 3.5) containing 5 mmol tetrabutylammonium phosphate and 13% methanol, with a flow rate of 1 mL/min and 190 nm wavelength adjustment. Samples were compared with the reference standards for reduced and oxidized glutathione obtained from Sigma-Aldrich Chemical Co. (St. Louis, MO, USA) [40].

Striatal and hippocampal MDA levels were quantified using the 250 × 4.5 mm Supelcosil LC-18 column (5 μm particle size and 80 A° pore size). The mobile phase consisting of 17.5:82.5 (v/v) methanol and 30 mM monobasic potassium phosphate (pH 3.6), with a flow rate of 1.5 mL/min and wavelength of 250 nm, was applied for detection. The MDA standard preparation involved dissolving 25 μL of 1,1,3,3 tetra-ethoxy-propane in 100 mL water to produce a 1 mM stock solution. The working standard was prepared by hydrolyzing 1 mL of 1,1,3,3-tetra-ethoxy-propane stock solution in 50 mL 1% sulfuric acid; the standard was then incubated for 2 h at room temperature. Sulfuric acid (1%) was used to dilute the resulting 20 nmol/mL MDA standard to obtain a final concentration of 1.25 nmol/mL, which served as the standard for measuring total MDA [41].

Nitric oxide striatal and hippocampal content was measured as the ratio of nitrite/nitrate (NO) following the method of Papadoyannis et al. [42]. A Hamilton PRP-X100 anion exchange HPLC column (4.1 × 150 mm, 10 µm) was used. A 45 to 55 volume ratio of 0.1 M sodium chloride and methanol was used as the mobile phase, with a 2 mL/min flow rate and 230 nm wavelength. A standard mixture of NOx was used to determine the separation of the peaks and retention times.

Tissue 8-OHdG level was determined using the method of Lodovici et al. [43]. The chromatographic separation used the C18 reverse phase column in series (Supelco, 5 pm, I.D. 0.46 × 25 cm) and an eluting solution of water:methanol (85:15 v/v) with 50 mM KH_2_PO_4_ (pH 5.5), employing a 0.68 mL/min flow rate, and adjusting the UV detector wavelength at 245 nm.

Energy markers (AMP, ADP, and ATP) were determined by injecting samples into a 15 × 0.4 cm Nucleosil C-18 column, using a mobile phase consisting of 1% methanol and 50 mM potassium phosphate (v/v, pH 5.5) at a 1 mL/min flow rate, and a UV detector wavelength of 210 nm [44].

Monoamines were determined after the samples were extracted using a solid phase extraction CHROMABOND column (NH2 phase Catalog No. 730031) from the trace elements and lipids. Then, samples were injected into an Aqua 5 μm C18 200 A°, 150 × 4.6 mm LC column (Phenomenex, USA), using 20 mM potassium phosphate (pH 2.7) as the mobile phase, at 1.5 mL/min flow rate and 290 nm UV wavelength [45]. Striatal and hippocampal GABA and glutamate levels were assessed by HPLC using the pre-column phenylisothiocyanate derivatization technique [46].

##### Determination of the Hippocampal Levels of the Neurotrophin, BDNF, and the Biomarkers of Neurodegeneration, α-Synuclein, Phosphorylated Tau and Beta-Amyloid 1–42

Hippocampal BDNF and α-synuclein levels were assessed using rat ELISA kits (Catalog No. E-EL-R1648 and E-EL-R1426, respectively) purchased from Elabscience Biotechnology Research (Houston, TX, USA). Rat ELISA kits were also used to determine the hippocampal levels of phosphorylated tau (Catalog No. ER1507, Wuhan Fine Biological Technology, Wuhan, China) and beta-amyloid 1–42 (Catalog No. LS-F26380, LifeSpan BioSciences, Seattle, Washington, USA). All kits adopted the sandwich ELISA technology and were used in accordance with the manufacturers’ instructions.

##### Determination of Striatal and Hippocampal Interleukin-6 Levels

The effect of HFD feeding for 24 weeks and its modulation by prebiotic and/or probiotic intervention on the inflammatory status was assessed by the determination of interleukin-6 (IL-6) levels in the rat striata and hippocampi. The assay was performed using a rat IL-6 ELISA kit (Catalog No. E-EL-R0015, Elabscience Biotechnology Research, Houston, TX, USA), employing the sandwich ELISA technique, following the manufacturer’s guidelines.

### Statistical Analysis

The normality of the data was tested by the Shapiro–Wilk test for normality. Data are displayed as the mean ± standard deviation (SD), and statistical comparisons between the means were conducted by one-way analysis of variance (ANOVA), followed by the Tukey or Duncan post hoc test. Statistical analyses were performed using GraphPad Prism 8 for Windows, GraphPad software, version 8.4.2 (Boston, MA, USA), and the SPSS 16 statistical package (SPSS Inc., Chicago, IL, USA). The differences among groups were considered statistically significant at *P* values of less than 0.05.

## Results

### *In vitro* Study Results

#### Changes in Viable Microbial Counts, pH, and GABA Level

Table 1 shows the microbial counts of the F-ASE and F-MRS. *L. plantarum* population grew significantly throughout the course of 24 h, after which they continued to increase, but much slower. No significant variation (*P* > 0.05) in the microbial growth of F-MRS was observed when the fermentation time was longer than 48 h. The initial counts in the F-ASE were found to be 8.36 ± 0.16 log CFU/mL. It was found that these counts increased significantly during fermentation, reaching 10.33 ± 0.10 log CFU/mL after 48 h, and then gradually stabilized throughout the duration of the fermentation process. This is likely due to the continuous nutrient depletion during the first 48 h of fermentation and the subsequent restriction of strain growth and reproduction. Yet, Asparagus extract is a complex mixture of certain minerals, proteins, dietary fibers, saponins, and flavonoids. Hence, other chemical components may be present during fermentation to influence the pH and cell count. Table 1 provides an illustration of the pH variations in F-ASE and F-MRS. The pH values dropped after fermentation. The pH values of F-ASE significantly declined from 6.63 ± 0.13 to 3.06 ± 0.02. This could be attributable to *L. plantarum* DMS 20174 producing SCFAs (lactic, propionic, and butyric acids) during the fermentation of ASE. The F-ASE samples had higher pH values, which may be due to the utilization of ASE prolonging the delay period and decreasing metabolism and microbiological viability.

**Table 1:** Viable counts (log CFU/mL), pH, and GABA (µg/mL), values obtained with *L. plantarum* DMS 20174 grown on MRS broth and asparagus extract, after 72 h of fermentation

Groups	Fermentation time (hr)	Viable counts (log CFU/mL)	pH	GABA (µg/mL)
F-MRS (Control)	**0**	8.53 ± 0.32 ^e^	6.65 ± 0.15 ^a^	-
	**24**	10.47 ± 0.14 ^bc^	4.02 ± 0.10 ^c^	0.682
	**48**	11.23 ± 0.06 ^a^	3.71 ± 0.10 ^d^	1.027
	**72**	11.57 ± 0.15 ^a^	2.97 ± 0.08 ^e^	1.310
F-ASE	**0**	8.36 ± 0.16 ^e^	6.63 ± 0.13 ^a^	-
	**24**	9.81 ± 0.45 ^d^	4.28 ± 0.06 ^b^	ND
	**48**	10.33 ± 0.10 ^c^	4.02 ± 0.08 ^c^	0.405
	**72**	10.84 ± 0.07 ^b^	3.06 ± 0.02 ^e^	0.979
*F-value*		85.22 ^***^	^***^	-

Data are presented as mean ± SD (*n* = 3) and were analyzed using One-Way ANOVA, followed by Duncan’s post hoc test. Different letter superscripts in the same column differ significantly (*P* < 0.05) in the viable counts or pH values among the levels of each fermentation parameter. *** *P* < 0.001. *F-MRS*, MRS medium was inoculated with *L. plantarum*, and incubated for 72 h at 37 °C (fermented MRS medium), serving as the control; *F-ASE*, *A. officinalis* extract was fermented for 72 h at 37 °C by *L. plantarum* (fermented Asparagus extract); *CFU*, colony-forming unit; *GABA*, gamma-aminobutyric acid; *ND*, not detected

Table 1 displays the GABA levels for the F-ASE and F-MRS. As the fermentation process continued, the GABA rate rose. The maximum increment of 1.310 µg/mL was observed in the MRS medium fermented for 72 h. This is because the MRS medium contains glucose, which is used to promote cell growth and the formation of lactic acid for LAB species [47]. The extracellular GABA was not detected in F-ASE after 24 h (Table 1), and it reached its peak after 72 h of fermentation. Asparagus species were regarded as acceptable raw materials for fermentation based on their nutritional and practical usefulness.

#### Reducing Power Assay

**Fig. 2:**
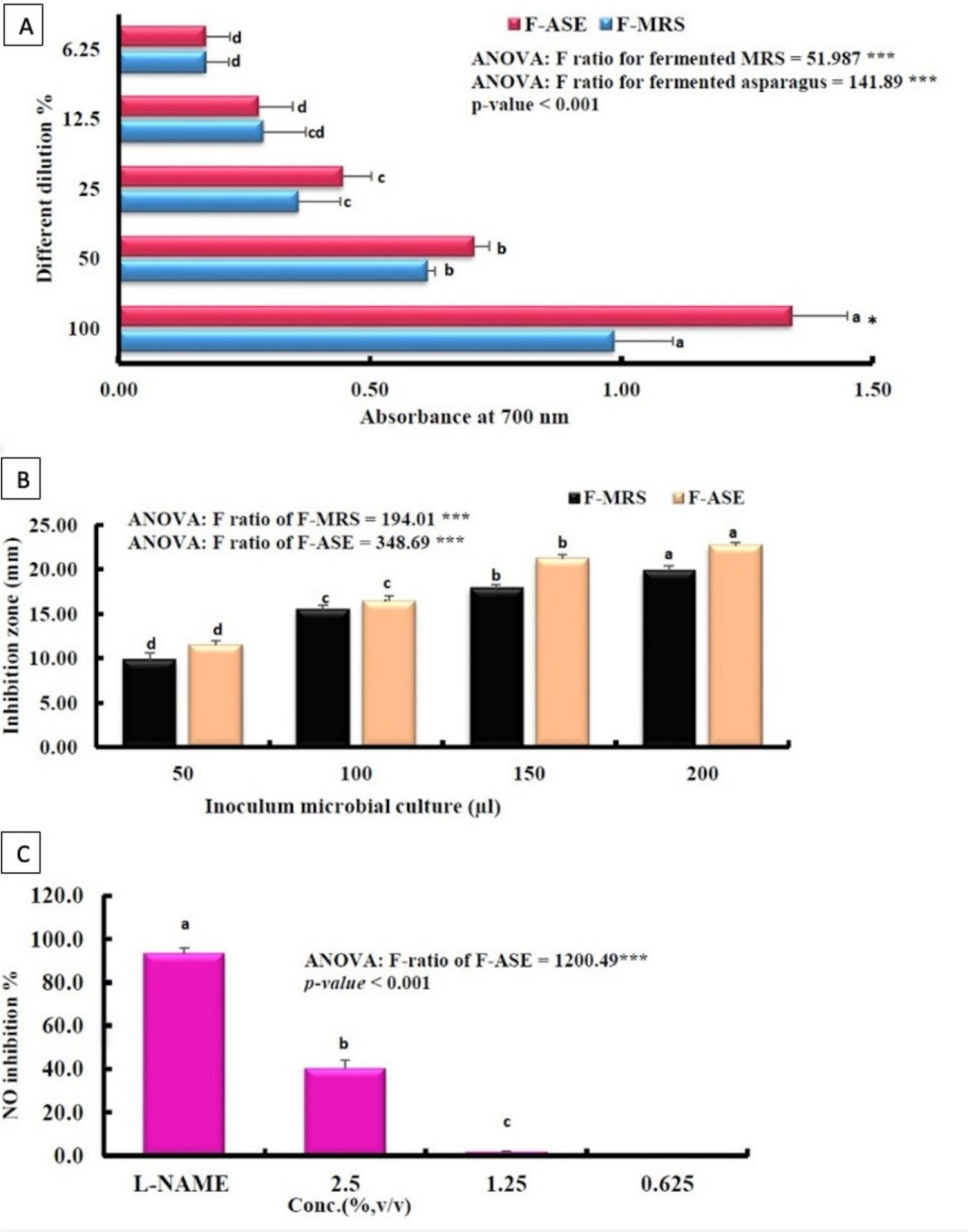
Reducing power capacity (**A**), bile salt hydrolase activity (**B**), and anti-inflammatory activity (**C**) of F-ASE. Data are presented as mean ± SD (*n* = 3) and were analyzed using One-Way ANOVA, followed by Duncan’s post hoc test. The different letters indicate significant differences between the different dilutions and/or inoculum microbial culture for each cell-free supernatant (CFS) of F-MRS or F-ASE at *P* < 0.05. * indicates significant difference between F-MRS and F-ASE for each dilution. F-ASE, fermented *Asparagus officinalis* extract; F-MRS, fermented MRS medium; L-NAME, Nω-Nitro-L-arginine methyl ester

Reducing power capacity is an important marker of antioxidant activity. Figure 2A displays the CFS-reducing power of F-MRS and F-ASE. Reducing power was found in both fermentative supernatants, and it was increased with concentration. The reducing power of F-ASE was higher than that of F-MRS; precisely when the dilution was 100%, the difference was significant (*P* < 0.05).

#### Bile Salt Hydrolase Activity

The growth of *L. plantarum* DMS 20174, which was grown previously on MRS and ASE, was observed on an agar plate containing bile salts and CaCl_2_, indicating its resistance to bile salts and the presence of BSH activity. The measured clear zones were between 9.83 ± 0.76 and 22.70 ± 0.30 mm (Fig. 2B). Because this strain is resistant to bile salts [47], this outcome was anticipated.

#### Inhibition of Nitric Oxide Production

In this study, the F-ASE ability to suppress NO generation was examined. In a conditioned medium, the LPS-stimulated cells produced substantial amounts of NO. When RAW cells were exposed to various F-ASE dilutions (0.625, 1.25, and 2.5% v/v), NO generation decreased dose-dependently. At 2.5%, the maximum inhibition (40.29%) was attained (Fig. 2C).

#### Metabolic Profiling of F-ASE and UF-ASE

The metabolic profile of *A. officinalis* extract before and after fermentation was examined for the first time. In order to determine whether flavonoids and saponins are present as potential contributors to their considerable biological activities, it is valuable to investigate the active chemicals in F-ASE and UF-ASE using an LC–MS-based approach. Table 2 contains a list of all the constituent peaks’ retention times and m/z values.

**Table 2:** Metabolite profiling of UF-ASE and F-ASE as analyzed by LC–MS/MS

Peak number	UF-ASE		F-ASE	
	*t*_R_	Base peak m/z	*t*_R_	Base peak m/z
Positive mode				
1	0.71	527.15	0.68	527.15
2	1.95	512.35	3.12	449.85^a^
3	3.13	449.80^a^	4.33	477.20
4	6.57	609.30^b^	4.76	437.15^c^
5	6.76	653.30	5.86	521.20^d^
6	6.91	697.35	6.30	565.25
7	8.31	1071.50	6.57	609.25
8	8.60	1057.50	6.76	653.35
9	11.15	775.35	6.91	697.35
10			7.05	741.35^e^
11			7.67	655.45
12			8.31	1071.50
13			8.61	1057.55
14			11.14	775.35
15			11.95	629.30
16			14.48	495.25
17			29.59	610.15^f^
Negative mode				
1	0.73	539.15	0.69	539.15
2	1.62	627.35	1.65	627.35
3	2.02	556.35	5.59	740.40
4	5.63	740.40	8.30	1047.45^ h^
5	5.94	537.30^i^	8.60	1033.45^j^
6	6.22	887.40^ k^	9.66	1029.45^ l^
7	8.32	1047.50^ h^	11.15	797.40^ m^
8	8.63	1033.50^j^	11.96	651.40
9	9.65	1029.45^ l^	14.50	913.40
10	11.15	797.40^ m^		
11	14.46	913.45		

Kaempferol glucoside^a^; diosmin^b^; Phloretin-2′-O-glucoside (Phlorizin)^c^; dehydrodiconiferyl alcohol-gamma′-O-glucoside^d^, robinin^e^, rutin^f^, protodioscin^h^; Olivil-4′-O-glucoside^i^, asparagoside F^j^; hydroxysarsapogenin^k^; pseudo-protodioscin^l^; dumoside^m^. *UF-ASE*, unfermented asparagus extract, without bacterial inoculum; *F-ASE*, *L. plantarum*–fermented *A. officinalis* extract; *LC*, liquid chromatography; *MS*, mass spectrometry; *tR*, retention time

**Fig. 3:**
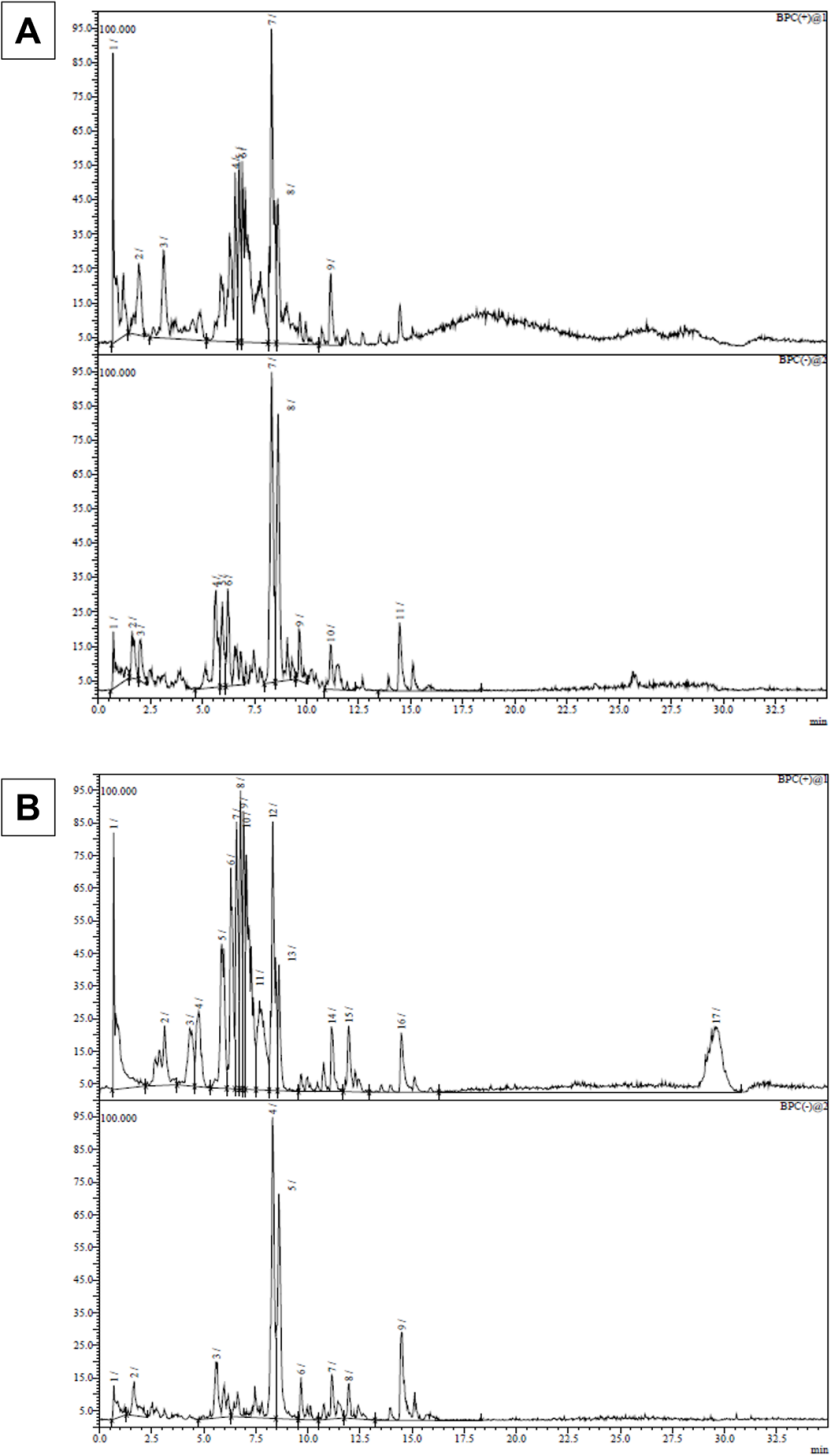
The LC–MS base-peak chromatograms of UF-ASE (**A**) and F-ASE (**B**) electrospray ionization (ESI) scan spectra in negative mode and in positive ion mode. The peaks are indicated with respective numbers in the list of compounds stated in Table 2. LC–MS, liquid chromatography–mass spectrometry; UF-ASE, unfermented *Asparagus officinalis* extract; F-ASE, fermented *Asparagus officinalis* extract

There were 11 and 9 peaks for UF-ASE and F-ASE, respectively, in the negative mode, compared to 9 and 17 unique base-peak chromatographs in the positive mode for those two fractions (Table 2 and Fig. 3). In comparison to UF-ASE, the positive mode showed better responsiveness and optimal profile of F-ASE ingredients. Both the UF-ASE and the F-ASE had some common constituent peaks. These changes were due to the fermentation, where many differences might be anticipated. While the remaining peaks have not been previously reported from microbial or plant origin and are reported for the first time, certain chemicals of Asparagus sp. were identified based on the prior literature (Table 2).

The retention periods and the MS spectral data of the separated peaks could indicate the potential existence of rutin (C_28_H_34_O_15_, peak number 17 at *t*_R_ 29.59, [M + 1] + m/z 610) and protodioscine (peaks 4, 7, at *t*_R_ 8.30 and 8.32, [M-1]- m/z 1047) in both fractions. Peaks 2 and 3 on the chromatograms correspond to kaempferol glucoside (C_27_H_30_O_15_), which showed a *t*_R_ of 1.95 and 3.13 min with [M + 1] + and a m/z value of 449 in positive mode.

With regard to the positive mode, peak 4, with a *t*_R_ of 4.76 min and m/z 437, was proposed to be phloretin-2’-O-glucoside (phlorizin). Peaks 4 and 7, with m/z 609 and molecular formula C_28_H_32_O_15_, were described as diosmin for UF-ASE and F-ASE, respectively. Finally, peak 5 was identified as dehydrodiconiferyl alcohol-gamma′-O-glucoside in F-ASE, whereas peak 10, with a *t*_R_ of 7.05 min, was characterized as robinin.

It was suggested that peak 5 in the negative mode, with a *t*_R_ of 5.94 min, was olivil-4′-O-glucoside. This compound was not detected in UF-ASE. Asparagoside F was identified in peaks 5 and 8 with m/z 1033 for the F-ASE and UF-ASE, respectively. Peaks 6 and 9 may be related to pseudo-protodioscin because they eluted at 9.66 and 9.65 min with m/z 1029, respectively. The molecule of peak 6 identified for UF-ASE has an aglycon of hydroxysarsapogenin and a *t*_R_ of 6.22 min. Peaks 7 and 10 produced a deprotonated substance that was identified as dumoside for both extracts at m/z 797. Our findings illustrated that F-ASE was more promising than UF-ASE, so it was examined for its anti-inflammatory activity.

### *In vivo* Study Results

#### Behavioral Study

##### Effect of Prebiotic and Probiotic Treatment, Alone or in Combination, on Spatial Memory in HFD-Fed Rats

**Fig. 4:**
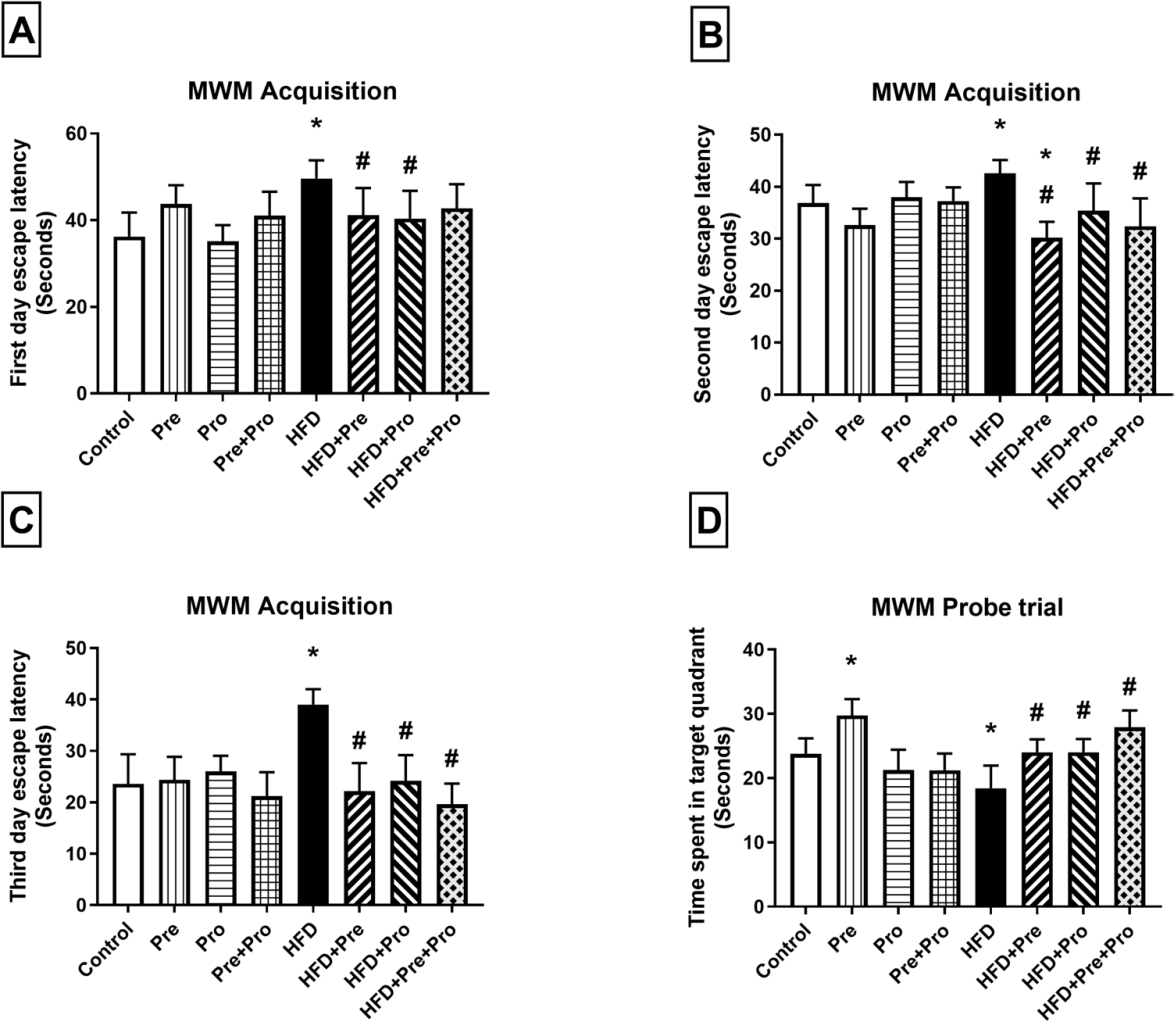
Effect of prebiotic, probiotic, and synbiotic treatment on spatial memory in HFD-fed rats. Spatial memory was assessed by the MWM test. Escape latencies were recorded on days 1, 2, and 3 in MWM acquisition trials (**A**–**C**). On day 4, retention memory was assessed in the MWM probe test (**D**). Data are presented as mean ± SD (*n* = 8) and were analyzed using One-Way ANOVA, followed by Tukey’s multiple comparisons test; * *P* < 0.05, significant difference versus the control group; # *P* < 0.05, significant difference versus the HFD group. HFD, high-fat diet; MWM, Morris water maze; Pre, prebiotic (Asparagus extract); Pro, probiotic (*Lactobacillus plantarum*)

Notable learning deficit was perpetrated by long-term HFD intake which significantly prolonged the escape latency by 37% (*P* < 0.0001), 15.5% (*P* < 0.05), and 65.3% (*P* < 0.01) on days 1, 2, and 3, respectively, in Morris water maze acquisition trials as compared to normally fed rats signifying learning impairment. Considerable improvement in cognitive function was achieved by prebiotic and probiotic treatment, either alone or in combination, evidenced by significantly reduced escape latencies in acquisition trials relative to untreated HFD-fed rats (17%, *P* < 0.05 and 18.5%, *P* < 0.01 lowered first day escape latency by the prebiotic and probiotic treatments, respectively; 29%, *P* < 0.0001, 17%, *P* < 0.01, and 24%, *P* < 0.0001 lowered second day escape latency by the prebiotic, probiotic, and combination, respectively; 43%, *P* < 0.001, 38%, *P* < 0.01, and 49.7%, *P* < 0.0001 lowered third day escape latency by the prebiotic, probiotic, and combination, respectively) (Fig. 4A–C).

The probe trial revealed significant retention memory deterioration by high-fat feeding, manifested by the 22.5% shorter time spent by HFD-challenged rats in the target quadrant as compared to the control group (*P* < 0.01). Retention memory was demonstrably restored by prebiotic and probiotic supplementations which significantly increased the time consumed in the target quadrant by 30.4%, in the case of single therapy (*P* < 0.01), and 51.5%, by the synbiotic combination (*P* < 0.0001) (Fig. 4D).

#### Microbial Colony Counts

Table 3 displays the effects of the probiotic, ASE, and their combination on the fecal microbiota of rats fed on the basal diet and HFD at the end of the experiment. Rats on the basal diet supplemented with ASE and *L. plantarum* had a considerable increase in total Lactobacilli (8.27 ± 0.38 log CFU/g) compared to the control group (6.98 ± 0.16 log CFU/g). *Lactobacillus* levels significantly dropped in the HFD group. In comparison to feeding only with HFD and normal basal diet, the probiotic-treated group had considerably higher levels of Lactobacilli, which led to a more stable change in the intestinal Lactobacilli population. However, no significant effect on total aerobic bacterial counts was recorded in all groups. Relative to the HFD group, rats treated with *L. plantarum* demonstrated a decrease in the number of Coliform bacteria and Staphylococci. All treatment groups had total Coliform counts that ranged from 4.07 ± 0.63 to 6.82 ± 0.33 log CFU/g and Staphylococci count that ranged from 5.52 ± 0.60 to 7.66 ± 0.22 log CFU/g. The amount of microscopic fungus in the colon of HFD was dramatically decreased by the probiotic strain (3.21 ± 0.21 log CFU/g) when compared to the HFD group (4.24 ± 0.09 log CFU/g).

**Table 3:** Colon microbial population (log CFU/g) of rats exposed to HFD and treated with ASE, *L. plantarum* or their combination

Group	Viable count (log CFU/g)				
	Total aerobes	*Lactobacillus* sp.	Coliform bacteria	Staphylococci	Fungi
Control	7.81 ± 0.71^a^	6.98 ± 0.16^ cd^	6.39 ± 0.21^ab^	7.59 ± 0.43^a^	4.30 ± 0.14^a^
Pre	7.45 ± 0.22^a^	7.37 ± 0.25^bc^	5.92 ± 0.26^bcd^	7.61 ± 0.12^a^	3.28 ± 0.20^b^
Pro	7.56 ± 0.29^a^	7.65 ± 0.14^b^	5.43 ± 0.33^ cd^	5.52 ± 0.60^b^	3.19 ± 0.20^bc^
Pre + Pro	7.39 ± 0.29^a^	8.27 ± 0.38^a^	4.07 ± 0.63^e^	5.73 ± 0.63^b^	2.83 ± 0.47^c^
HFD	8.06 ± 0.45^a^	6.71 ± 0.37^d^	6.82 ± 0.33^a^	7.51 ± 0.11^a^	4.24 ± 0.09^a^
HFD + Pre	8.13 ± 0.36^a^	7.20 ± 0.09^bcd^	6.03 ± 0.47^bc^	7.66 ± 0.22^a^	3.30 ± 0.07^b^
HFD + Pro	7.82 ± 0.48^a^	7.52 ± 0.31^bc^	5.34 ± 0.15^d^	5.64 ± 0.49^b^	3.21 ± 0.21^bc^
HFD + Pre + Pro	7.61 ± 0.62^a^	7.68 ± 0.59^b^	4.22 ± 0.09^e^	5.65 ± 0.34^b^	2.96 ± 0.16^bc^
*F-value*	**1.06**	6.52 ***	23.52 ***	19.18 ***	18.26 ***

Data are presented as mean ± SD (*n* = 8) and were analyzed using One-Way ANOVA, followed by Duncan’s post hoc test. ^a,b,c^Mean values within a column with unlike superscript letters are significantly different (*P* < 0·05). *** *P* < 0.001. *HFD*, high-fat diet; *Pre*, prebiotic (Asparagus extract); *Pro*, probiotic (*Lactobacillus plantarum*); *CFU*, colony-forming unit

#### Translocation of Lactobacilli

Total Lactobacilli were plated on samples from the spleen, liver, and kidney. The absence of translocation was indicated by the samples having no Lactobacilli growth. Lactobacilli supplemented in diet, either singly or combined, were safe and did not translocate to other internal organs, since none of the probiotic- or synbiotic-treated groups displayed any adverse translocation of Lactobacilli to the organs.

#### Biochemical Investigations

##### Effect of Prebiotic and Probiotic Treatment, Alone or in Combination, on Serum Leptin Level and Lipid Profile in HFD-Fed Rats

**Fig. 5:**
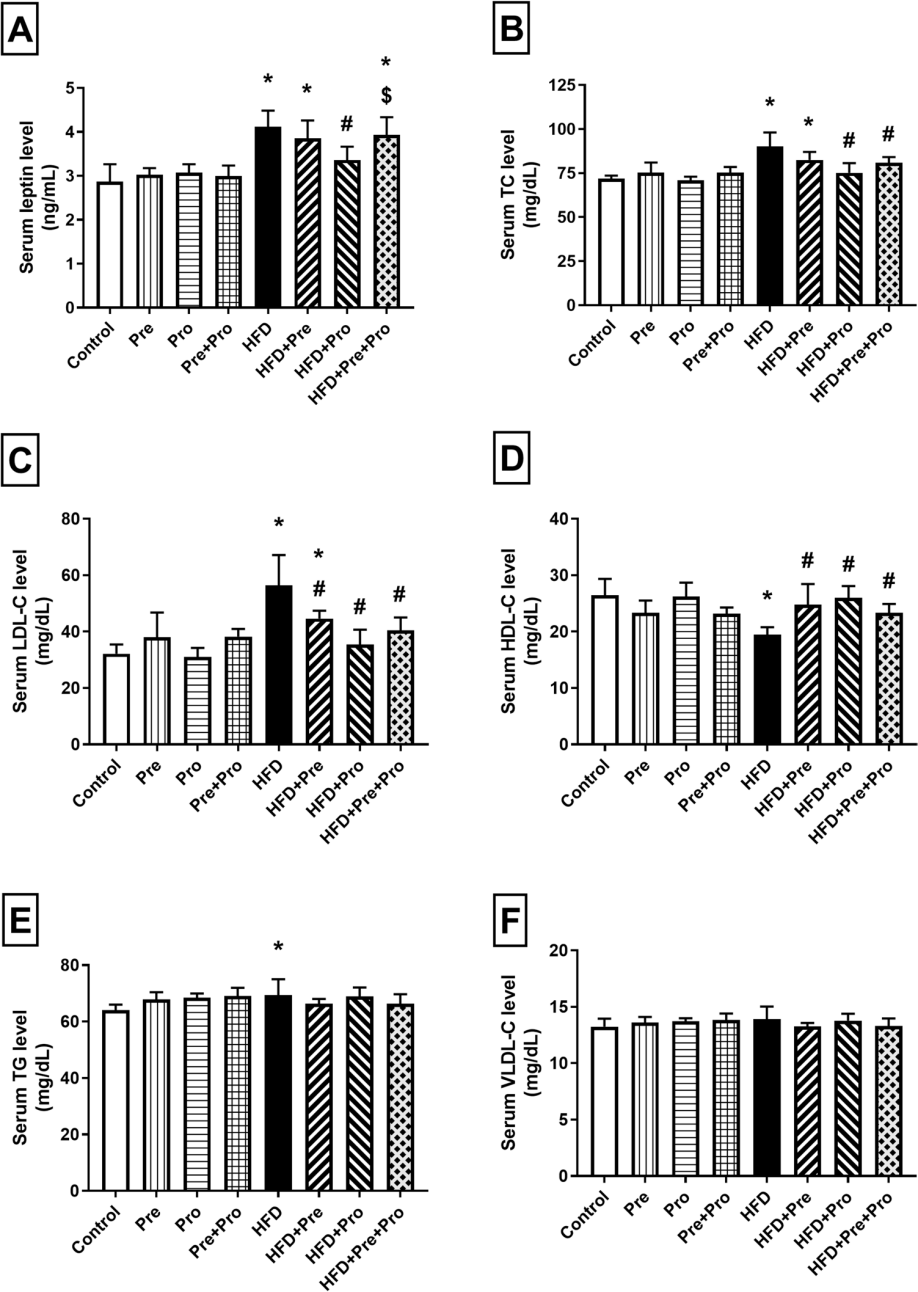
Effect of prebiotic, probiotic, and synbiotic treatment on serum leptin level and lipid profile in HFD-fed rats. (**A**) Serum leptin level. (**B**) Serum total cholesterol level. (**C**) Serum low-density lipoprotein cholesterol level. (**D**) Serum high-density lipoprotein cholesterol level. (**E**) Serum triglyceride level. (**F**) Serum very low-density lipoprotein cholesterol level. Data are presented as mean ± SD (*n* = 6) and were analyzed using One-Way ANOVA, followed by Tukey’s multiple comparisons test; * *P* < 0.05, significant difference versus the control group; # *P* < 0.05, significant difference versus the HFD group; $ *P* < 0.05, significant difference versus the HFD + Pro group. HFD, high-fat diet; Pre, prebiotic (Asparagus extract); Pro, probiotic (*Lactobacillus plantarum*); TC, total cholesterol; LDL-C, low-density lipoprotein cholesterol; HDL-C, high-density lipoprotein cholesterol; TG, triglyceride; VLDL-C, very low-density lipoprotein cholesterol

Serum leptin level was significantly elevated by HFD intake when compared to normally fed rats (43.5%, *P* < 0.0001) and was only restored to normal by the probiotic treatment which significantly decreased its level by 18.4% (*P* < 0.001**)** relative to the HFD-fed rats (Fig. 5A).

HFD intake resulted in significant alterations in the serum lipid profile as manifested by 25.3% and 75.4% higher TC and LDL-cholesterol levels, respectively, and 26.3% lower HDL-cholesterol level (*P* < 0.0001) compared to the normal control group. Such alterations were significantly mitigated by the three tested interventions with effective normalization achieved by the probiotic (*P* < 0.0001 for TC, HDL, and LDL levels) and the prebiotic/probiotic combination (*P* < 0.05 for TC and HDL, *P* < 0.001 for LDL level). Serum TG level was significantly elevated in HFD-fed rats (9% elevation, *P* < 0.05) compared to the control; however, only apparent non-statistically significant improvement was observed due to the tested treatments. No significant variation was observed in serum VLDL level in any of the study groups. Prebiotic treatment also significantly restored serum HDL level (*P* < 0.01) and significantly lowered serum LDL level by about 21% (*P* < 0.05) compared to the HFD group (Fig. 5B–F).

##### Effect of Prebiotic and Probiotic Treatment, Alone or in Combination, on Striatal and Hippocampal Neurotransmitter Alterations in HFD-Fed Rats

**Fig. 6:**
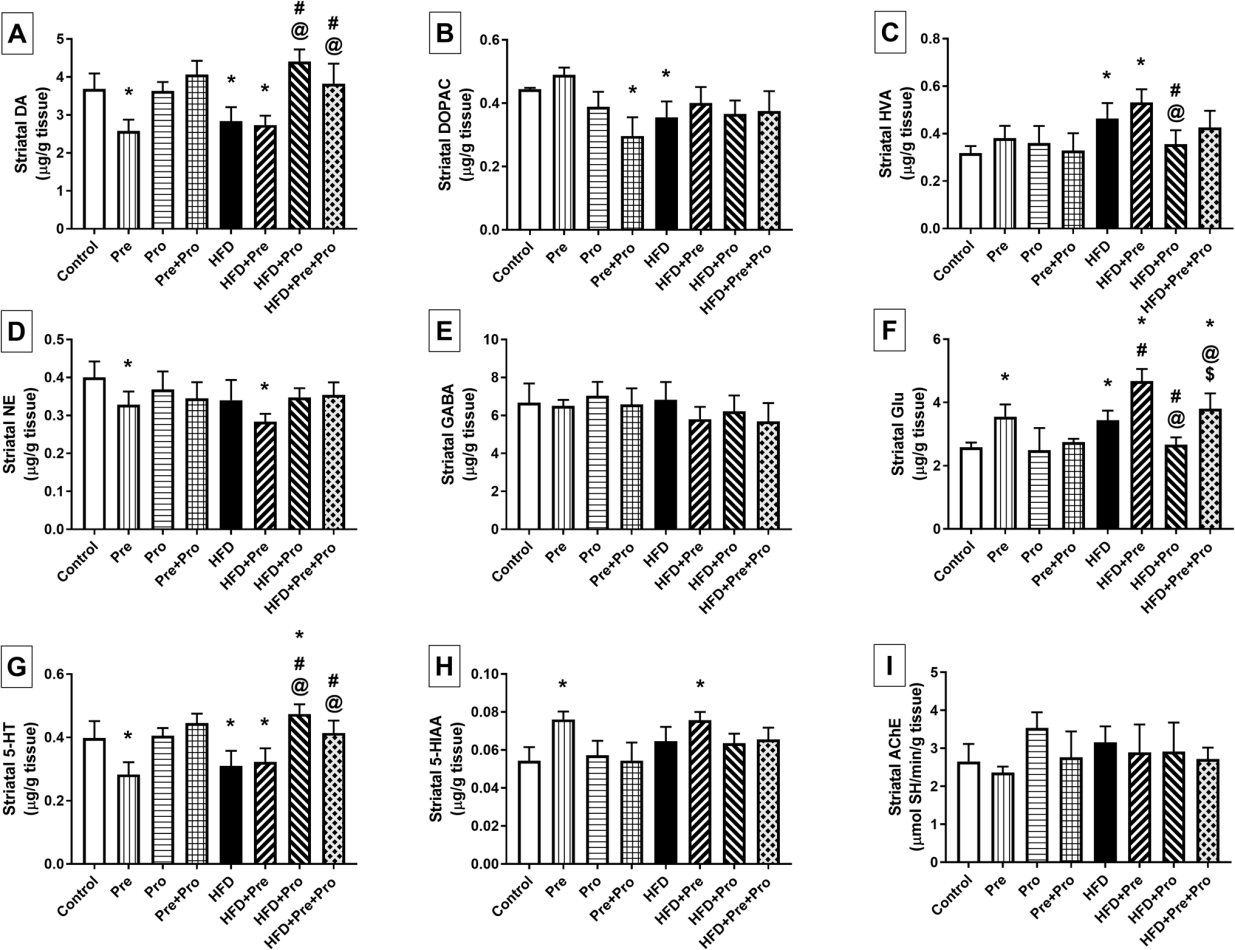
Effect of prebiotic, probiotic, and synbiotic treatment on striatal levels of neurotransmitters and their metabolites in HFD-fed rats. (**A**) Striatal dopamine level. (**B**) Striatal dihydroxyphenylacetic acid level. (**C**) Striatal homovanillic acid level. (**D**) Striatal norepinephrine level. (**E**) Striatal gamma-aminobutyric acid level. (**F**) Striatal glutamate level. (**G**) Striatal serotonin level. (**H**) Striatal 5-hydroxyindoleacetic acid level. (**I**) Striatal acetylcholinesterase activity. Data are presented as mean ± SD (*n* = 6) and were analyzed using One-Way ANOVA, followed by Tukey’s multiple comparisons test; * *P* < 0.05, significant difference versus the control group; # *P* < 0.05, significant difference versus the HFD group; @ *P* < 0.05, significant difference versus the HFD + Pre group; $ *P* < 0.05, significant difference versus the HFD + Pro group. HFD, high-fat diet; Pre, prebiotic (Asparagus extract); Pro, probiotic (*Lactobacillus plantarum*); DA, dopamine; DOPAC, 3,4-dihydroxyphenylacetic; HVA, homovanillic acid; NE, norepinephrine; GABA, gamma-aminobutyric acid; Glu, glutamate; 5-HT, 5-hydroxytryptamine (serotonin); 5-HIAA, 5-hydroxyindoleacetic acid; AChE, acetylcholinesterase

**Fig. 7:**
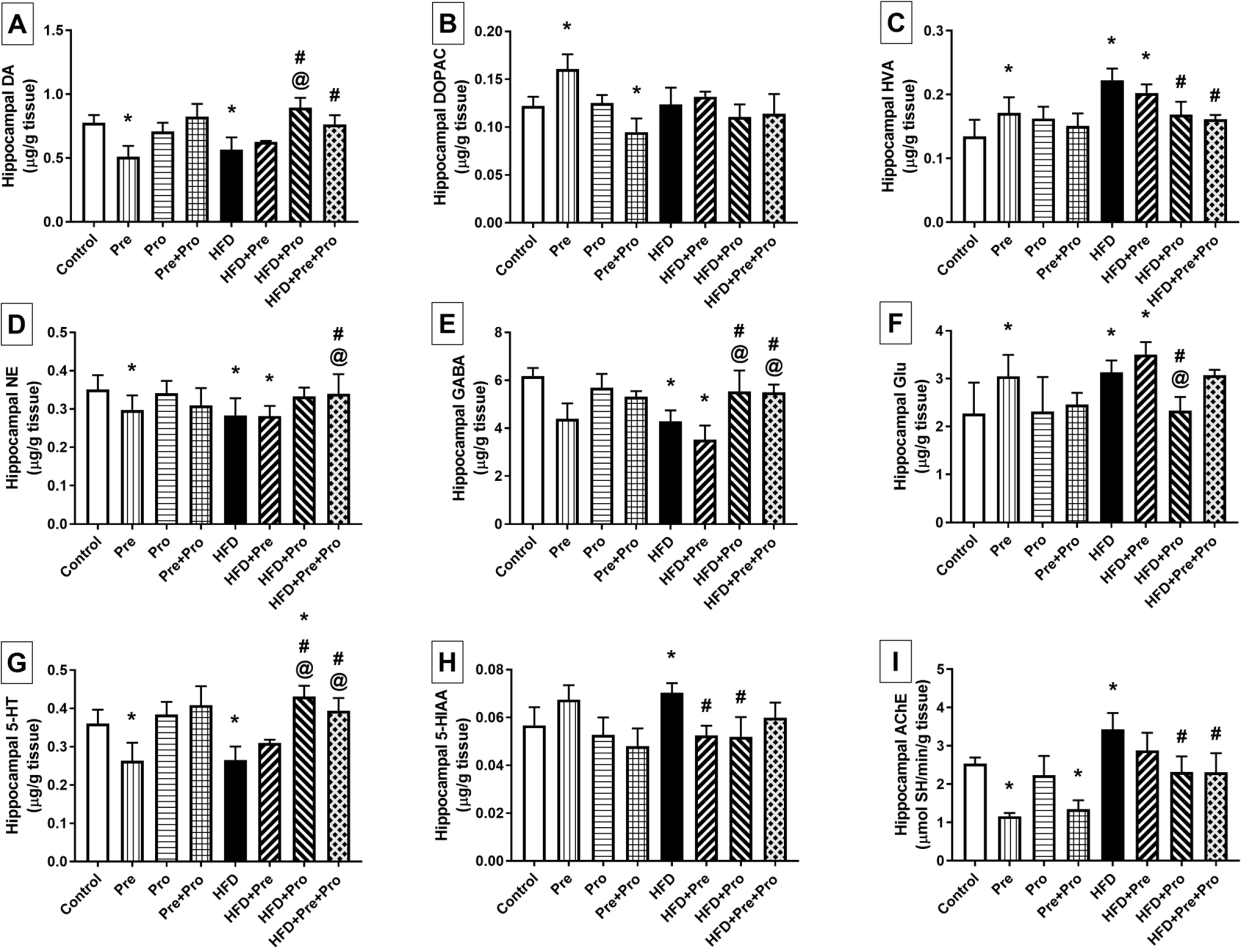
Effect of prebiotic, probiotic, and synbiotic treatment on hippocampal levels of neurotransmitters and their metabolites in HFD-fed rats. (**A**) Hippocampal dopamine level. (**B**) Hippocampal dihydroxyphenylacetic acid level. (**C**) Hippocampal homovanillic acid level. (**D**) Hippocampal norepinephrine level. (**E**) Hippocampal gamma-aminobutyric acid level. (**F**) Hippocampal glutamate level. (**G**) Hippocampal serotonin level. (**H**) Hippocampal 5-hydroxyindoleacetic acid level. (**I**) Hippocampal acetylcholinesterase activity. Data are presented as mean ± SD (*n* = 6) and were analyzed using One-Way ANOVA, followed by Tukey’s multiple comparisons test; * *P* < 0.05, significant difference versus the control group; # *P* < 0.05, significant difference versus the HFD group; @ *P* < 0.05, significant difference versus the HFD + Pre group. HFD, high-fat diet; Pre, prebiotic (Asparagus extract); Pro, probiotic (*Lactobacillus plantarum*); DA, dopamine; DOPAC, 3,4-dihydroxyphenylacetic; HVA, homovanillic acid; NE, norepinephrine; GABA, gamma-aminobutyric acid; Glu, glutamate; 5-HT, 5-hydroxytryptamine (serotonin); 5-HIAA, 5-hydroxyindoleacetic acid; AChE, acetylcholinesterase

Figures 6 and 7 illustrate the changes observed in striatal and hippocampal neurotransmitter levels, respectively. HFD feeding inflicted significant reduction in striatal and hippocampal DA (23% and 26.9%, respectively, *P* < 0.05) and 5-HT levels (22.5%, *P* < 0.01 and 25%, *P* < 0.001, respectively), as well as in hippocampal GABA level (30.9%, *P* < 0.0001). Such decrements were significantly reverted by both the probiotic (55.3% and 56.1% increments, respectively, *P* < 0.0001 in DA levels, 51.6% and 59.3% increments, respectively, *P* < 0.0001 in 5-HT levels, and 29.2% increment in GABA level, *P* < 0.01) and the prebiotic/probiotic combination (34.9% and 33.3% increments in DA levels, respectively, *P* < 0.05, 28.5% increment in GABA level, *P* < 0.05, and 32.3% and 48.1% increments in 5-HT levels, respectively, *P* < 0.0001). A significant decrease in hippocampal NE was detected in HFD-fed rats (20%, *P* < 0.01) that was revoked only by the combined treatment which boosted the NE level by 21.4% relative to that of the HFD group (*P* < 0.05).

On the other hand, significant 33% (*P* < 0.01) and 38.1% (*P* < 0.05) increments were observed in striatal and hippocampal glutamate levels, respectively, compared to control values, and were significantly averted by the probiotic treatment as shown by 22.4% (*P* < 0.01) and 25.7% (*P* < 0.05) lower striatal and hippocampal glutamate levels, respectively, in the probiotic-treated group than in the HFD-fed group.

Furthermore, HFD intake incited a marked 35.6% increase in hippocampal AChE esterase activity compared to normal rats (*P* < 0.01), and this effect was significantly abrogated by the probiotic (*P* < 0.0001) and the prebiotic/probiotic combination (*P* < 0.001), both achieving about 33% reduction in its activity when compared with that of the HFD group.

The HFD-induced decline in dopamine levels, in response to the 24-week high-fat dietary regimen, was paralleled by significant 46% (*P* < 0.001) and 66% (*P* < 0.0001) elevations in the striatal and hippocampal levels of the dopamine metabolite homovanillic acid (HVA), respectively. The striatal level was reinstated by the probiotic treatment reducing it to a 23.5% significantly lower value (*P* < 0.05) compared to that of the HFD-fed group. On the other hand, the hippocampal HVA level was effectively normalized by both the probiotic and the prebiotic/probiotic combination (*P* < 0.001), showing 24.3% and 27.4% lower values compared to the HFD group level. HFD-induced dysregulation of DA metabolism was also revealed by deranged striatal 3,4-dihydroxyphenylacetic acid (DOPAC) levels showing significant 20% reduction compared to controls (*P* < 0.05). The effect of HFD consumption on striatal DOPAC levels was apparently counteracted by all tested interventions, albeit not reaching statistical significance, resulting in values that did not vary significantly from the controls.

In parallel with the HFD-evoked alterations in 5-HT levels, a significant 24.1% elevation in the serotonin metabolite 5-HIAA was observed in the hippocampi of HFD-challenged rats compared to the controls (*P* < 0.05). This effect was significantly annulled by prebiotic and probiotic single therapies which resulted in 25.4% (*P* < 0.01) and 26% (*P* < 0.001) decrements, respectively, compared to the HFD-fed group. On the other hand, an apparent non-significant 19% increase in striatal 5-HIAA level was detected in HFD-exposed rats relative to the controls.

##### Effect of Prebiotic and Probiotic Treatment, Alone or in Combination, on Neuronal Alterations in HFD-Fed Rats

**Fig. 8:**
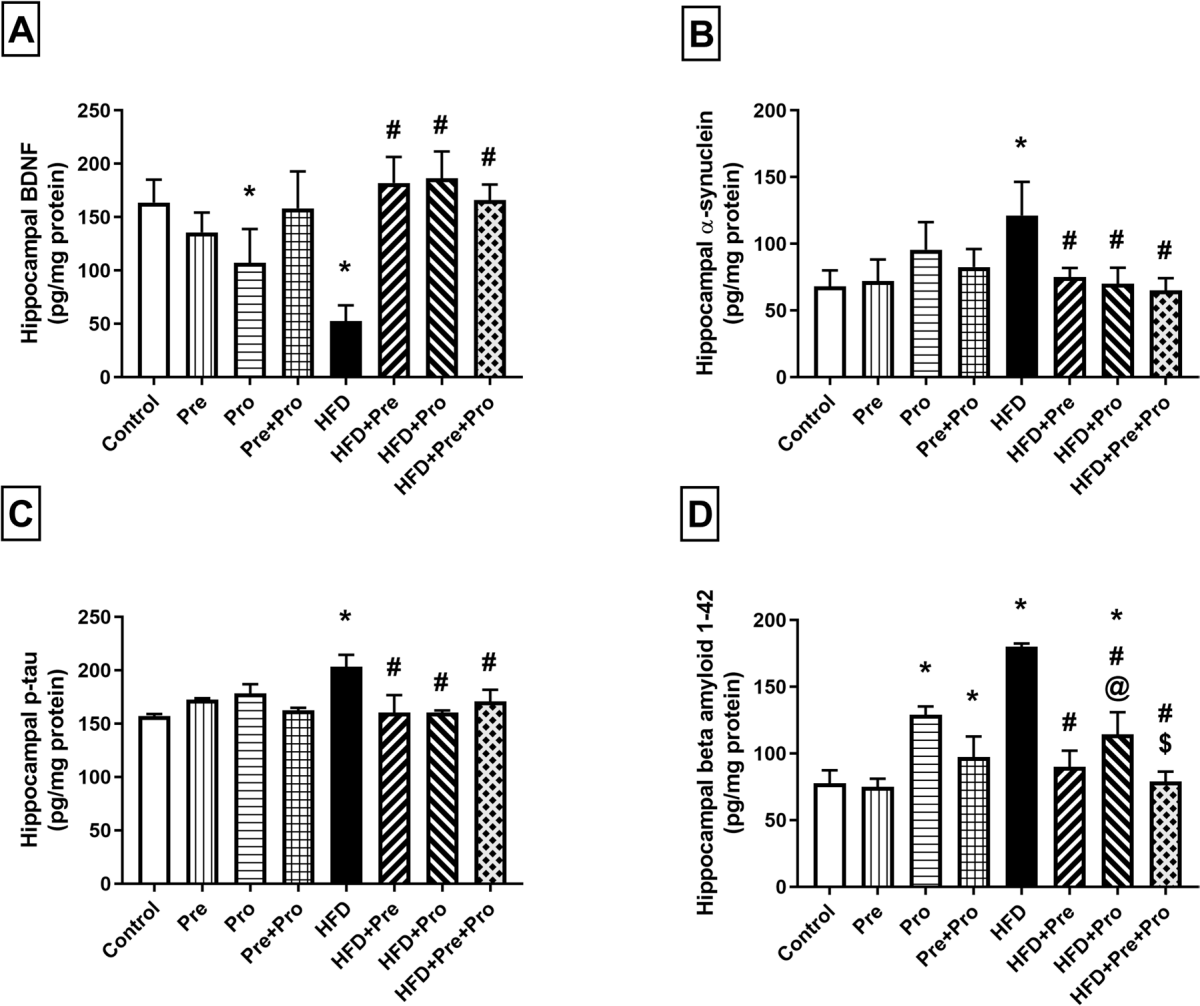
Effect of prebiotic, probiotic, and synbiotic treatment on hippocampal levels of neurodegenerative markers in HFD-fed rats. (**A**) Hippocampal brain-derived neurotrophic factor level. (**B**) Hippocampal alpha-synuclein level. (**C**) Hippocampal phosphorylated tau level. (**D**) Hippocampal beta-amyloid 1–42 level. Data are presented as mean ± SD (*n* = 6) and were analyzed using One-Way ANOVA, followed by Tukey’s multiple comparisons test; * *P* < 0.05, significant difference versus the control group; # *P* < 0.05, significant difference versus the HFD group. HFD, high-fat diet; Pre, prebiotic (Asparagus extract); Pro, probiotic (*Lactobacillus plantarum*); BDNF, brain-derived neurotrophic factor; p-tau, phosphorylated tau

Rats fed the HFD displayed a dramatic 67.8% decline (*P* < 0.0001) in hippocampal BDNF content that was effectively reinstated by all the tested treatments, as demonstrated by values reaching 3.5-fold (*P* < 0.0001) in the prebiotic- and probiotic-treated groups, and 3.1-fold (*P* < 0.0001) in the combination-treated group compared to the HFD level (Fig. 8A). On the other hand, HFD-fed rats’ hippocampi showed a considerable 78% increase in α-synuclein level (*P* < 0.0001), a marked 29.3% elevation in p-tau content (*P* < 0.0001), and a substantial upsurge of amyloid beta 1–42 (Aβ_42_) content resulting in a 2.3-fold value (*P* < 0.0001) compared to the control group (Fig. 8B–D). Such HFD-imposed alterations were all counteracted by prebiotic and probiotic treatment, alone and in combination. Compared to the HFD-exposed group, significant 38% (*P* < 0.01), 42% (*P* < 0.01), and 46.4% (*P* < 0.0001) decrements in α-synuclein levels were attained by prebiotic, probiotic, and synbiotic treatment, respectively (Fig. 8B). Refurbished p-tau levels were manifested by significant 21% and 16% reduction (*P* < 0.0001) achieved by single and combined therapy, respectively, compared to HFD-challenged rats (Fig. 8C). Recuperated Aβ_42_ levels were demonstrated by 50.1%, 36.5%, and 56.1% lower values (*P* < 0.0001) in rats treated with ASE, *L. plantarum* and their combination, respectively, relative to the corresponding values in untreated HFD-fed rats (Fig. 8D).

##### Effect of Prebiotic and Probiotic Treatment, Alone or in Combination, on Striatal and Hippocampal Oxidative Status in HFD-Fed Rats

**Fig. 9:**
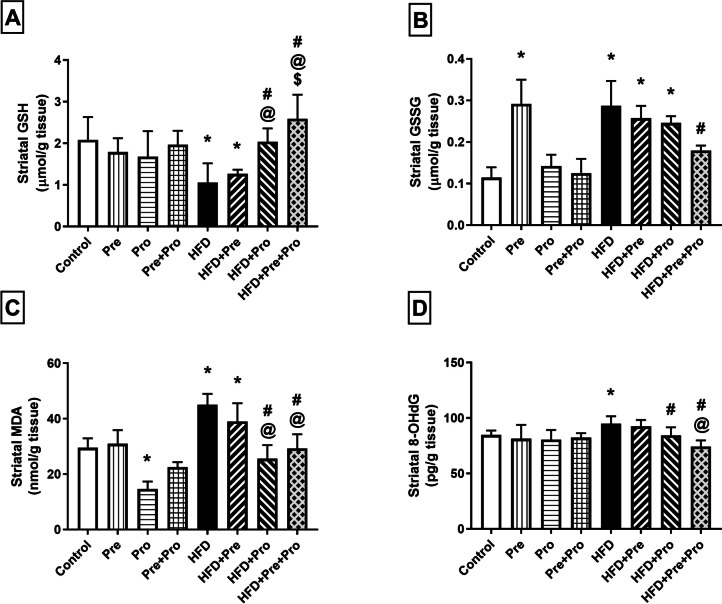
Effect of prebiotic, probiotic, and synbiotic treatment on striatal oxidative status in HFD-fed rats. (**A**) Striatal reduced glutathione level. (**B**) Striatal oxidized glutathione level. (**C**) Striatal malondialdehyde level. (**D**) Striatal 8-hydroxy-2′-deoxyguanosine level. Data are presented as mean ± SD (*n* = 6) and were analyzed using One-Way ANOVA, followed by Tukey’s multiple comparisons test; * *P* < 0.05, significant difference versus the control group; # *P* < 0.05, significant difference versus the HFD group; @ *P* < 0.05, significant difference versus the HFD + Pre group; $ *P* < 0.05, significant difference versus the HFD + Pro group. HFD, high-fat diet; Pre, prebiotic (Asparagus extract); Pro, probiotic (*Lactobacillus plantarum*); GSH, reduced glutathione; GSSG, oxidized glutathione; MDA, malondialdehyde; 8-OHdG, 8-hydroxy-2′-deoxyguanosine

**Fig. 10:**
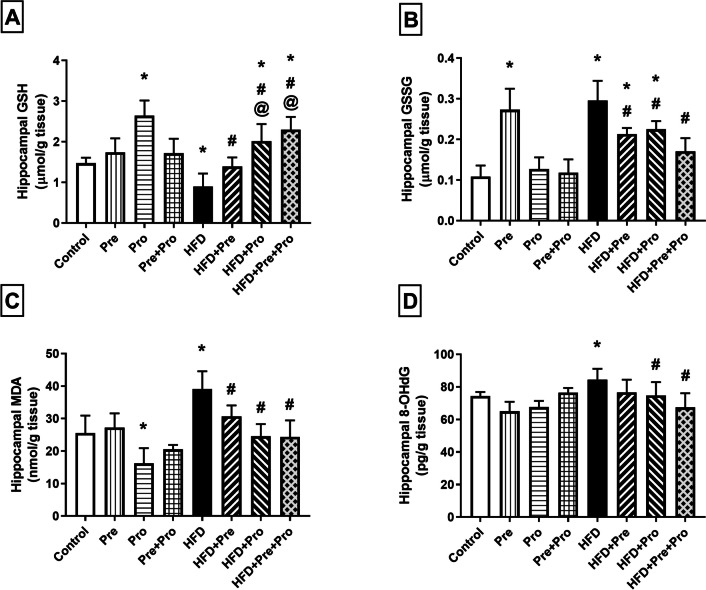
Effect of prebiotic, probiotic, and synbiotic treatment on hippocampal oxidative status in HFD-fed rats. (**A**) Hippocampal reduced glutathione level. (**B**) Hippocampal oxidized glutathione level. (**C**) Hippocampal malondialdehyde level. (**D**) Hippocampal 8-hydroxy-2′-deoxyguanosine level. Data are presented as mean ± SD (*n* = 6) and were analyzed using One-Way ANOVA, followed by Tukey’s multiple comparisons test; * *P* < 0.05, significant difference versus the control group; # *P* < 0.05, significant difference versus the HFD group; @ *P* < 0.05, significant difference versus the HFD + Pre group. HFD, high-fat diet; Pre, prebiotic (Asparagus extract); Pro, probiotic (*Lactobacillus plantarum*); GSH, reduced glutathione; GSSG, oxidized glutathione; MDA, malondialdehyde; 8-OHdG, 8-hydroxy-2′-deoxyguanosine

Figures 9 and 10 display the detected striatal and hippocampal alterations, respectively, in the oxidative status of the different studied groups. HFD intake instigated substantial oxidative stress in the rat striata and hippocampi, indicated by significant depletion of striatal and hippocampal content of reduced glutathione (GSH) compared to normally fed rats (49.1%, *P* < 0.0001 and 39%, *P* < 0.01, respectively). Striatal GSH content was significantly reinstated by probiotic as well as prebiotic/probiotic combined treatment achieving 92.6% and 144.8% higher levels, respectively, relative to that of HFD rats (*P* < 0.0001). Hippocampal GSH content was significantly ameliorated by the prebiotic treatment which effectively normalized its level (54.4% replenishment, *P* < 0.05), and was boosted to above the normal level by probiotic treatment as well as by the prebiotic/probiotic combination resulting in 2.2- and 2.6-fold GSH levels, respectively, compared to that of the HFD group (*P* < 0.0001) (Figs. 9A and 10A).

Both striatal and hippocampal contents of oxidized glutathione (GSSG) were significantly elevated in HFD-fed rats which showed 2.5- and 2.7-fold levels, respectively, compared to normally fed rats (*P* < 0.0001). Treatment with either the prebiotic or the probiotic significantly decreased the hippocampal GSSG level (28% and 23.9% decrements, respectively, *P* < 0.01), but only apparently decreased the striatal level where the decrement did not reach statistical significance when compared with HFD-fed rats. On the other hand, combined prebiotic/probiotic treatment effectively normalized the GSSG levels in both striatal and hippocampal tissues attaining 37.5% (*P* < 0.01) and 42.2% (*P* < 0.0001) decrements, respectively, compared to the HFD group values (Figs. 9B and 10B).

Feeding rats with the HFD elicited a notable enhancement in striatal and hippocampal lipid peroxidation, evidenced by significantly heightened levels of malondialdehyde (MDA), the end product of lipid peroxidation, compared to the corresponding levels in normally fed rats, as manifested by 52.7% (*P* < 0.01) and 53.2% (*P* < 0.001) increments, respectively. Probiotic treatment alone or combined with the prebiotic achieved substantial alleviation of the striatal and hippocampal MDA alterations (43.2%, *P* < 0.0001 and 35%, *P* < 0.001 lowering of striatal MDA level by probiotic and combination, respectively, and about 37%, *P* < 0.0001 lowering of hippocampal MDA level by both probiotic and combination) which were effectually reversed by such treatments. Moreover, hippocampal MDA was significantly reinstated by the prebiotic treatment resulting in a 21.7% (*P* < 0.05) lower value compared to that of the HFD group (Figs. 9C and 10C).

High-fat-fed rats exhibited significant 12.2% and 13.5% elevations in striatal and hippocampal 8-hydroxy-2′-deoxyguanosine (8-OHdG) levels (*P* < 0.05), respectively, compared to the control group. Such HFD-provoked 8-OHdG insult was effectively rectified by probiotic supplementation resulting in 11.3% and 11.6% significantly lower striatal and hippocampal levels in the probiotic-treated group (*P* < 0.05), respectively, than in the HFD-challenged group. The remedial effect of probiotic treatment was augmented when combined with prebiotic administration, as demonstrated by a 22% and 20.1% significantly lower striatal and hippocampal levels in the synbiotic-treated group (*P* < 0.0001), respectively, compared to those in the untreated HFD-exposed rats. On the other hand, single prebiotic treatment failed to achieve any significant improvement in the elevated 8-OHdG in either of the two brain regions (Figs. 9D and 10D).

##### Effect of Prebiotic and Probiotic Treatment, Alone or in Combination, on Striatal and Hippocampal Inflammatory Status in HFD-Fed Rats

**Fig. 11:**
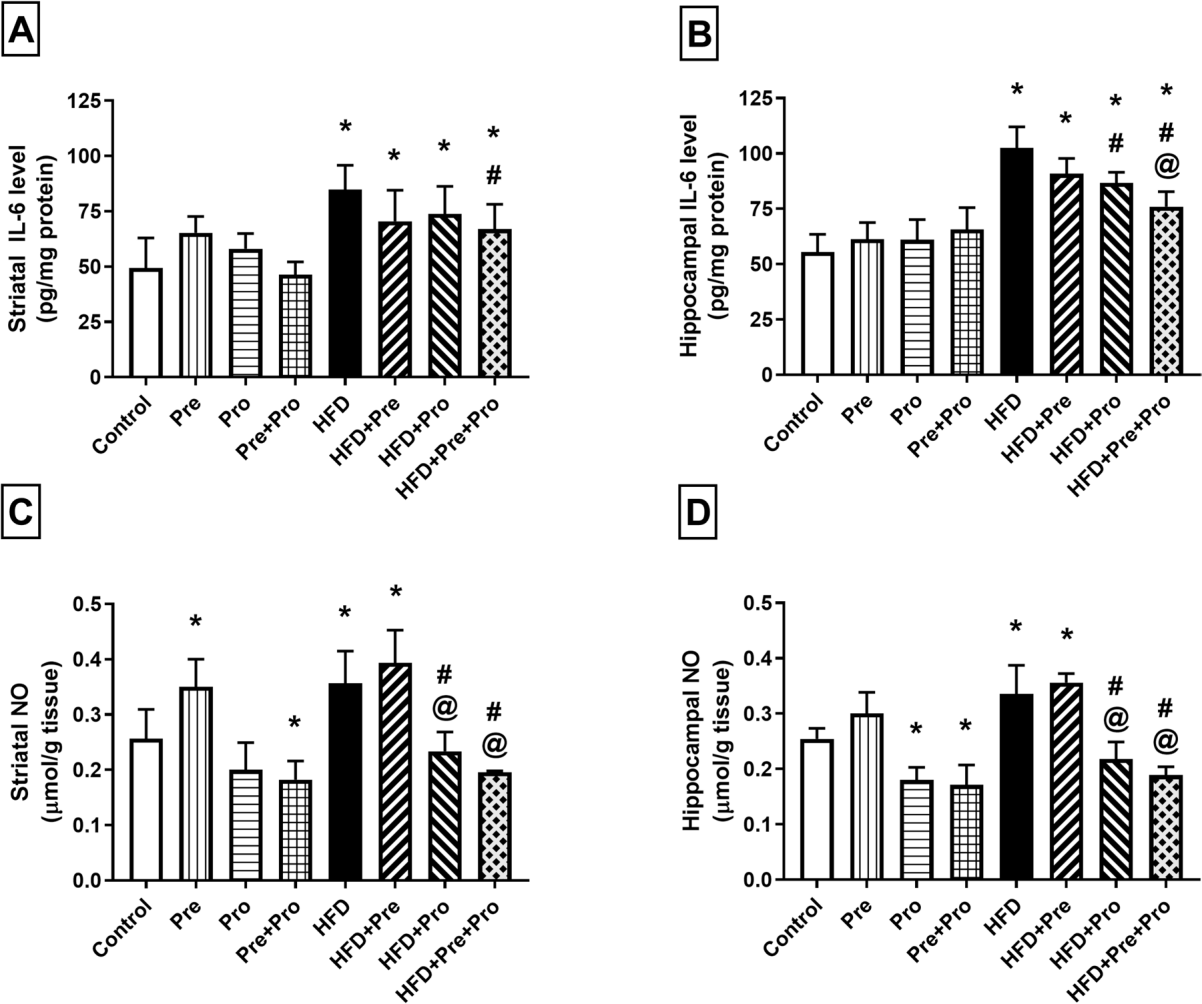
Effect of prebiotic, probiotic, and synbiotic treatment on striatal and hippocampal inflammatory status in HFD-fed rats. (**A**) Striatal interleukin-6 level. (**B**) Hippocampal interleukin-6 level. (**C**) Striatal nitric oxide level. (**D**) Hippocampal nitric oxide level. Data are presented as mean ± SD (*n* = 6) and were analyzed using One-Way ANOVA, followed by Tukey’s multiple comparisons test; * *P* < 0.05, significant difference versus the control group; # *P* < 0.05, significant difference versus the HFD group; @ *P* < 0.05, significant difference versus the HFD + Pre group. HFD, high-fat diet; Pre, prebiotic (Asparagus extract); Pro, probiotic (*Lactobacillus plantarum*); IL-6, interleukin-6; NO, nitric oxide

High-fat feeding for 24 weeks incited a 71.8% spike in striatal IL-6 content (*P* < 0.0001), compared to the control group. Although neither ASE nor *L. plantarum* single therapy could significantly curb this IL-6 elevation, their synbiotic combination attained a significant 21% reduction in striatal IL-6 level (*P* < 0.05), compared to HFD-exposed rats (Fig. 11A). In the hippocampus, IL-6 level was substantially increased to reach 1.85-fold (*P* < 0.0001) in HFD-fed rats, compared to the normal control group. While prebiotic therapy, alone, failed to alleviate the IL-6 upsurge, probiotic therapy, alone and combined with prebiotic supplementation, significantly lowered the hippocampal IL-6 level by 15.3% (*P* < 0.01) and 25.75% (*P* < 0.0001), respectively relative to the untreated HFD-challenged group (Fig. 11B).

Significant elevations in striatal and hippocampal NO contents were also observed in HFD-fed rats compared to the normal control group (39.5%, *P* < 0.001 and 31.8%, *P* < 0.01, respectively). These HFD-induced NO alterations were significantly attenuated by probiotic treatment alone or combined with the prebiotic resulting in normalized NO levels. This was manifested by 34.5% and 45.2% reduction in striatal levels as well as 35% and 43.7% mitigation in hippocampal levels by probiotic treatment and prebiotic/probiotic combination, respectively (*P* < 0.0001) (Fig. 11C, D).

##### Effect of Prebiotic and Probiotic Treatment, Alone or in Combination, on Striatal and Hippocampal Energy Status in HFD-Fed Rats

**Fig. 12:**
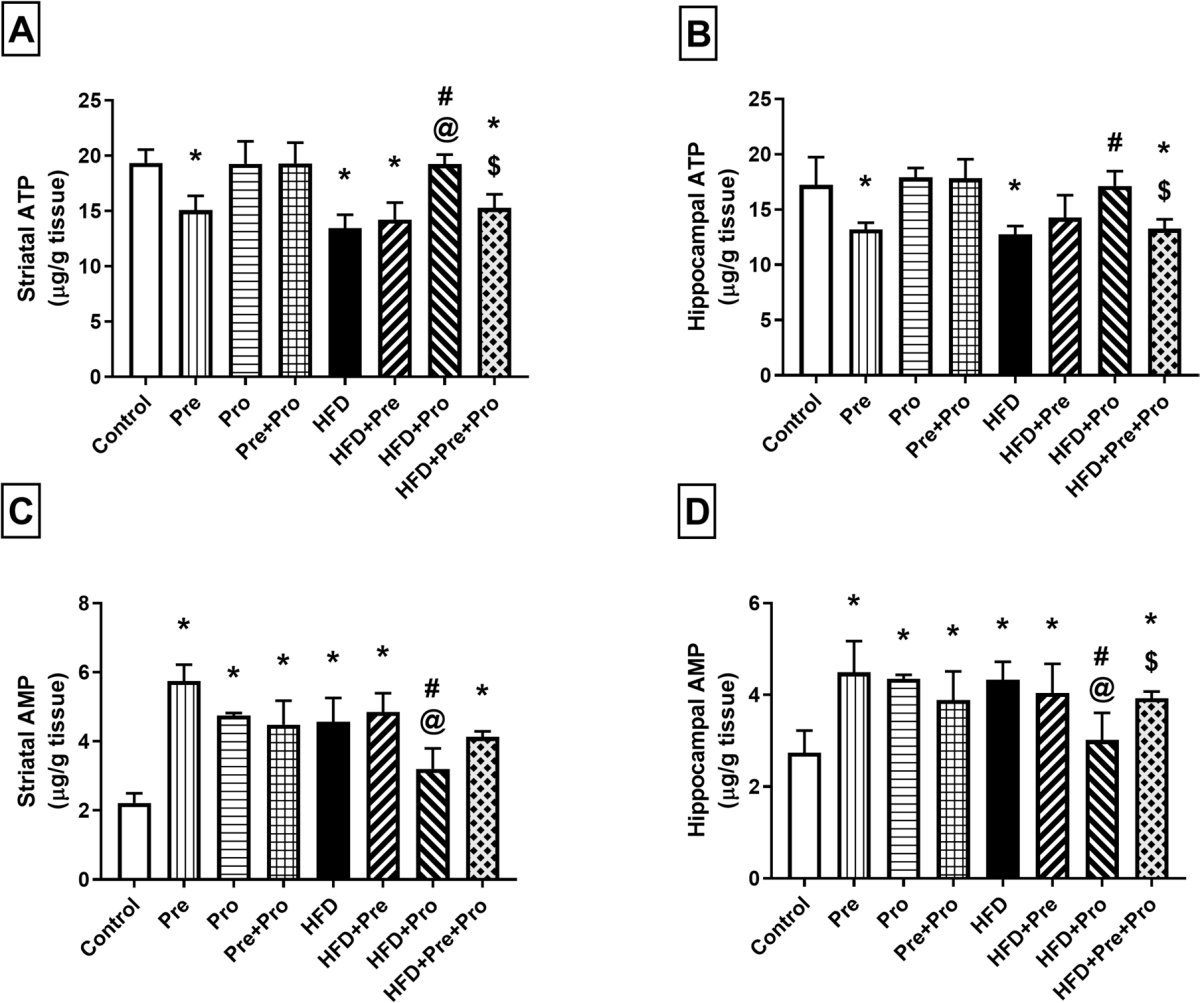
Effect of prebiotic, probiotic, and synbiotic treatment on striatal and hippocampal energy status in HFD-fed rats. (**A**) Striatal adenosine triphosphate level. **(B**) Hippocampal adenosine triphosphate level. (**C**) Striatal adenosine monophosphate level. (**D**) Hippocampal adenosine monophosphate level. Data are presented as mean ± SD (*n* = 6) and were analyzed using One-Way ANOVA, followed by Tukey’s multiple comparisons test; * *P* < 0.05, significant difference versus the control group; # *P* < 0.05, significant difference versus the HFD group; @ *P* < 0.05, significant difference versus the HFD + Pre group; $ *P* < 0.05, significant difference versus the HFD + Pro group. HFD, high-fat diet; Pre, prebiotic (Asparagus extract); Pro, probiotic (*Lactobacillus plantarum*); ATP, adenosine triphosphate; AMP, adenosine monophosphate

A comparable pattern of alterations was observed in the brain energy markers ATP and AMP, where HFD-fed rats exhibited significant reduction in striatal and hippocampal ATP levels (30.5%, *P* < 0.001 and 26%, *P* < 0.01, respectively) coupled with a significant increase in the corresponding AMP levels (106% and 58.1%, respectively, *P* < 0.0001) relative to control rats. Such energy deficit was significantly revamped only by the probiotic treatment, as demonstrated by 43.1% (*P* < 0.001) and 34.3% (*P* < 0.01) replenishment of striatal and hippocampal ATP levels, respectively, and about 30% decrease (*P* < 0.0001) in both striatal and hippocampal AMP levels compared to the respective values in the HFD group (Fig. 12).

## Discussion

The present study investigated the ameliorative effects of the probiotic *L. plantarum* DMS 20174, prebiotic asparagus extract (ASE), and their synbiotic combination against cognitive impairment and neuronal degeneration in HFD-fed rats. HFD intake resulted in dyslipidemia and hyperleptinemia, along with Aβ accumulation, tau hyperphosphorylation, α-synuclein elevation, and BDNF diminishment in the hippocampus, in addition to neurotransmitter and redox imbalance, heightened inflammation, and energy decline in the striatum and hippocampus with subsequent cognitive dysfunction. These impairments are possibly mediated through changes in gut microbiota. Daily consumption of probiotic (*L. plantarum* DMS 20174), prebiotic (ASE), or synbiotic for 12 weeks markedly alleviated these HFD-induced aberrations.

Consistent with previous reports [1, 4, 5], the present study showed that HFD consumption significantly deteriorated cognitive function in rats, as demonstrated by the impaired Morris water maze performance. These observations indicate the deleterious impact of long-term intake of high-fat diet on the rats’ spatial learning and memory. Excessive dietary fat has been reported to instigate hippocampal-hypothalamic neuronal apoptosis, leading to hippocampal atrophy which is an important cause of cognitive dysfunction and memory impairment.

In the current investigation, a substantial upsurge of Aβ_42_ and a marked elevation in p-tau content were observed in the hippocampi of rats fed on HFD. Long-term dietary consumption of high fat can promote the cleavage of β-amyloid precursor protein (APP) by proteolytic cleavage enzymes, such as β-secretase and γ-secretase, into various amino acid fragments, eventually yielding the Aβ40 and Aβ42 fragments [48]. The accretion of sizable insoluble amyloid fibrils results in amyloid plaque formation and dissemination through the brain. The dense plaque buildup in the hippocampus, amygdala, and cerebral cortex may trigger microglia and astrocyte activation, in addition to axonal, dendrite, and synaptic loss, with ensuing cognitive perturbations [49]. Tau, a microtubule-associated protein, is a key player in the assemblage and stabilization of microtubules, and in various cellular processes [50]. In the context of neuronal functioning, tau is implicated in neuronal signaling and synaptic plasticity [51]. It has been hypothesized that the dissemination of amyloid-β-driven synaptic destruction throughout the axon incites tau phosphorylation, its consequent detachment from microtubules, and tangle formation. By virtue of the microtubules’ supportive function for the axon, the detachment of aberrant hyperphosphorylated tau from microtubules provokes axonal damage [52]. Accordingly, a deleterious upshot of aberrant amyloid-β and abnormally phosphorylated tau formation is the compromised synaptic function and axonal integrity, and, thus, amyloid-β upsurge and tau hyperphosphorylation are both well-documented hallmarks of AD [49]. Furthermore, we also observed a marked elevation in hippocampal α-synuclein levels in HFD-fed rats. α-Synuclein, a protein that is abundantly expressed in the brain, is believed to play multiple roles including the orchestration of neurotransmitter release. The aggregation of misfolded α-synuclein is an integral contributor to neurodegenerative pathologies by virtue of its major role in amyloid-β and tau fibrillization [53]. Hence, the observed overexpression of α-synuclein could be involved in the corresponding neurotransmitter imbalance, amyloidopathy, and tauopathy perpetrated by high fat intake. In fact, α-synuclein accumulation has been reported to inflict several synucleinopathies among which are Parkinson’s disease, dementia with Lewy bodies, and multiple system atrophy [1]. Together, the observed spikes in hippocampal β-amyloid, α-synuclein, and phosphorylated tau expression in the HFD-fed group are significant indices of neurodegeneration.

In addition to the observed surge in the hippocampal markers of neurodegeneration, we found that HFD intake demonstrably lowered hippocampal BDNF levels. Hampering BDNF signaling can restrain synaptic plasticity and curb hippocampal neurogenesis, thereby impairing hippocampal‐dependent learning and memory [2], which implies that the fall in hippocampal BDNF may possibly play a role in the cognitive impairment brought on by high-fat consumption in rats. In alignment with our findings, many investigations indicate that HFD downregulates BDNF mRNA and protein expression levels, which are associated with an increased susceptibility to memory deficits [54, 55]. Oxidative stress, sparked by ROS generation, is presumably implicated in BDNF decline, thereby contributing to defective cognition [54]. In light of this justification, the hippocampal glutathione and lipid peroxidation alterations observed in our study could account, at least partly, for the corresponding changes in hippocampal BDNF levels which, in turn, might explain the respective cognitive modifications.

Compelling data point out the implication of cerebral oxidative perturbations, especially in the hippocampus, in the deterioration of cognitive function [56, 57]. In accordance with previous studies [58–60], we found that high-fat dietary intake for 24 weeks promoted substantial oxidative stress in the rat brain. The oxidative insult was revealed by significantly elevated levels of striatal and hippocampal malondialdehyde, signifying enhancement of lipid peroxidation, as well as significant depletion of striatal and hippocampal content of reduced glutathione, indicative of endogenous antioxidant exhaustion. Robust evidence derived from both human and animal models indicates a close connection between oxidative stress, Aβ accumulation, and aberrant tau phosphorylation. Specifically, p-tau directly influences complex I activity, which aids in Aβ-driven disruption of mitochondrial function and generation of ROS [58]. Simultaneously, excessive levels of ROS have been shown to promote the formation and accretion of Aβ fibrils, tau phosphorylation, and death of neuronal cells. This is explained on the basis that the membrane-associated oxidative tension sparked by Aβ deregulates ceramide and cholesterol metabolism, eliciting a neurodegenerative cascade culminating in further Aβ buildup and tau hyperphosphorylation [59]. Collectively, these data point to a vicious cycle that may gradually worsen the course of the disease and ultimately cause neuronal death. This cycle involves mitochondrial function derangement, enhanced oxidative distress, depleted antioxidant mechanisms, and overproduction of Aβ and p-tau, further perturbing mitochondrial functioning and ROS generation [58]. Considering the aforesaid interplay between oxidative stress, amyloidopathy, and tauopathy, the currently observed HFD-induced oxidative insult could be linked to the corresponding Aβ_42_ and p-tau alterations.

In parallel with prior research [60], the present investigation revealed a heightened state of cerebral inflammation in the HFD-exposed rats as demonstrated by discernibly raised striatal and hippocampal IL-6 concentrations. IL-6 reportedly mediates the pro-inflammatory responses to numerous stimuli, both systemically and in the brain [60]. Concurring with the HFD-provoked inflammation, the results of the present study showed elevations in striatal and hippocampal NO contents in HFD-fed rats. Among the most crucial inflammatory mediators is the NO radical that is synthesized from L-arginine by nitric oxide synthase (NOS). It plays a part in a myriad of biological processes. When cells are activated by LPS and cytokines, inducible nitric oxide synthase (iNOS) generates high amounts of NO, a process that is further linked to the production of powerful reactive species, among which is the peroxynitrite radical. It could thus be assumed that the elevated levels of NO, associated with long-term HFD feeding, are the result of iNOS upregulation in the striatal and hippocampal tissue. This presumption is supported by a former study reporting that 12-week HFD intake inflicted iNOS upregulation in the rat hippocampus [61]. Furthermore, Aβ stimulates the production of NO by upregulating the expression of iNOS, which is essential for the chain of events that results in cell death [62]. In light of the aforesaid justifications, the oxidative and NO alterations observed in the current study could be linked to the corresponding changes in Aβ_42_ levels. Therefore, cognitive impairment, observed in this study in response to HFD intake in rats, could be, at least partially, ascribed to the concurrently witnessed hippocampal oxidative and inflammatory insults [57].

In the current study, HFD-fed rats showed a striatal and hippocampal energy deficit as evidenced by a marked decrease in ATP levels and a considerable increase in the corresponding AMP levels relative to control rats. The majority of cellular reactions in the brain are powered by free energy that is generated in mitochondria through the aerobic oxidation of glucose. The dysregulation of tau and APP proteins seen in response to HFD intake was hypothesized to have an impact on mitochondrial processes and to alter the availability of energy. In neurons with β-amyloid accretion, essential enzymes in the mitochondrial metabolic chain are halted, causing damage to the respiratory chain and a reduction in ATP synthesis [63]. Furthermore, synaptic energy deficit is caused by tau hyperphosphorylation, which induces mitochondrial abnormalities [64]. Synaptic protein synthesis may be impacted by the defective energy status at the synapses [59].

In conformity with former investigations [65, 66], the present findings showed that HFD-fed rats recorded dyslipidemia and hyperleptinemia. Dyslipidemia has been associated with increased susceptibility to Alzheimer’s pathology in elderly adults through exacerbating cognitive debility [67]. Hypercholesterolemia in cholesterol-fed rabbits has been linked to reduced hippocampal and cortical volumes [68]. Moreover, posterior cingulate gray matter volumes and verbal memory have been deleteriously impacted by elevated levels of LDL-C [69], whereas raised levels of HDL-C confer defense against AD and hippocampal atrophy [66]. The accumulation of adipose tissue in HFD-fed rats may have caused abnormal adipokine production and secretion, which in turn may have contributed to the observed hyperleptinemia [70]. Obesity-incited decline in leptin responsiveness, known as leptin resistance, causes adipocytes to produce more leptin and results in hyperleptinemia as a way for the body to make up for the dampened leptin responsiveness [65]. It is interesting to note that because leptin is implicated in an array of neuropathological events, such as amyloidogenesis, tau hyperphosphorylation, neuroinflammation, oxidative stress, disrupted synaptic and cognitive function, it may be integral in the crosstalk between metabolic status and neurological ailments [71].

In accordance with past investigations, our findings revealed that high fat consumption disturbed the balance of amino acid and monoamine neurotransmitters, which may be related to the learning and cognitive impairment observed in this study [72, 73]**.** In the current study, HFD-fed rats showed lower hippocampal and striatal 5-HT levels compared to normal control rats. The hippocampal 5-HT perturbations align with previous research demonstrating HFD-induced decline in extracellular 5-HT levels in the murine hippocampus [72]. Our results also revealed an elevation in the main serotonin metabolite, 5-hydroxyindoleacetic acid (5-HIAA), in the hippocampi of the rats fed on HFD, suggesting a high rate of serotonin turnover [73]. Excessive activation of monoamine oxidase, the key enzyme for 5-HT catabolism, is a possible explanation for such neurochemical changes [73]. The herein reported findings also demonstrated that chronic HFD consumption caused dysregulation of the dopaminergic system, as manifested by the decrease of DA levels and increase in the concentrations of HVA, the main DA metabolite, in the striatum and hippocampus. The high concentration of the DA metabolite, HVA, suggests a high rate of DA breakdown [74]. It has been postulated that elevated HVA levels disrupt mitochondrial function, thereby impairing ATP production, which might account for the energy deficit we observed in HFD-fed rats. The raised HVA level could also contribute to excessive ROS generation, subsequently aggravating oxidative stress and the ensuing inflammation and apoptosis [74]. The current observations are in line with those of Ma and co-workers who observed a drop in striatal DA content in rats on an HFD regimen for a 13-week duration [75]. Numerous former investigations revealed dopaminergic level decline and DA receptor downregulation in other brain limbic regions. Relevant reports indicated that DA levels in the nucleus accumbens are diminished in a variety of animal models of obesity and high fat intake [76, 77], which could be attributed to mitigated stimulation of DA release and reduced vesicle size [76].

The GABA-glutamate imbalance, witnessed in the present investigation, in response to high fat consumption is in harmony with Sickmann and co-workers who reported disruptions in the hippocampal GABA-glutamate-glutamine cycle in obese rats [78]. The significant increase in hippocampal glutamate noted in the current study is supported by the results of Fritz and colleagues showing that mice fed on an HFD have a focally extended excitatory postsynaptic current, probably due to declined glutamate buffering [77]. Substantially increased brain glutamate signaling sparks multifarious cytotoxic events leading to lipid peroxidation and damage of vital structures such as neuronal lipid membranes, proteins, and DNA, thereby contributing to the oxidative, inflammatory, and neurodegenerative ramifications seen in the HFD-fed rats. Moreover, pursuant to Tian and co-workers’ reported anti-inflammatory effect of GABA in mice fed with high-fat diets [79], the currently observed GABA decline can exacerbate the inflammatory condition associated with high-fat dietary intake. In the current study, the HFD-fed group had higher AChE activity than the control group. AChE decomposes acetylcholine, a cholinergic neurotransmitter in the brain-nerve system; hence, excessive AChE release may lead to cognitive dysfunction [80].

Cognition and behavior can be modulated by gut microbiota via the microbiota-gut-brain axis [4, 6]. Chronic HFD intake reportedly provokes gut dysbiosis through boosting the growth of Proteobacteria, that primarily comprise Gram-negative lipopolysaccharide-containing bacteria, in the gut [81] and disrupting the integrity of the gut barrier by the repression of tight junction proteins [82]. This “leaky gut” enables the translocation of luminal LPS and LPS-containing bacteria from the gut lumen to the lamina propria wherein the innate immune cells are actuated, thereby sparking an inflammatory response [81]. Furthermore, high-fat dietary intake has lately been demonstrated to increase blood–brain barrier permeability [83], thereby exposing the brain to a multitude of deleterious entities. The cognitive deficit could also be aggravated by high brain exposure to pro-inflammatory cytokines, including IL-1β, IL-6, and TNF-α [4, 6]. Moreover, many studies point out that the abundance of certain bacterial species in the intestine might impact Aβ deposition predisposing to AD development [84, 85].

In parallel with former reports [81], our results demonstrated that long-term HFD consumption led to gut dysbiosis by enhancing the growth of Coliform bacteria and decreasing the *Lactobacillus* count. The present study showcased the ability of probiotic and prebiotic treatments to favorably modulate the gut microbial composition. It was also found that *L. plantarum*, compared to ASE, seems to play an effective role in raising *Lactobacillus* species and reducing Coliform and Staphylococci bacteria as well as fungi populations. It is noteworthy that synbiotic supplementation, compared to separate supplementation, had a more substantial role in reducing Coliform bacteria as well as increasing the *Lactobacillus* population.

Using the Morris water maze task, the current research findings revealed that *L. plantarum* and ASE interventions, either separately or in combination, alleviated learning and memory impairment in rats fed on the HFD. Our findings concur with previous investigations in which *L. plantarum* NDC 75017 conferred significant relief in aged rats with D-galactose-induced mitochondrial damage and learning and memory-associated derangements [23]. Furthermore, the results of the current research demonstrated that long-term administration of *L*. *plantarum*, ASE, or their combination attenuated the elevations in amyloid-β, p-tau, and α-synuclein and replenished the level of BDNF in the hippocampi of HFD-fed rats. Moreover, elevated hippocampal AChE activities, associated with high-fat intake, have also been reinstated by the treatment of HFD-fed rats with *L*. *plantarum.* It is important to emphasize that synbiotic administration was associated with more noticeable improvement compared to either *L. plantarum* or ASE treatments alone. Consistent with our results, it was found that oral treatment with *L. plantarum* C29-fermented soybean (DW2009) in an AD murine model attenuated memory deficits and inhibited amyloid-β expression by controlling gut microbiota composition and boosting BDNF expression [14]. In another study conducted by Chunchai and co-workers [86], the intake of probiotics (*L. piracies* HII01), prebiotics (xylooligosaccharide), and synbiotics for 12 weeks ameliorated hippocampal plasticity and oxidative stress, and improved memory and learning deficits in rats given a high-fat diet. They reported that these changes were associated with modifications in gut microbiota.

The present study demonstrated that *L. plantarum* therapy had the potential to recuperate the endogenous antioxidant capacity, to hamper lipid peroxidation and 8-OHdG formation, and to curtail the IL-6 and NO upsurge in both studied brain regions, indicating that probiotics could effectively reduce brain oxidative stress and inflammation. The antioxidant capability of *Lactobacillus*-based probiotic therapy is in context with Chen and co-workers’ report that oral treatment of type 2 diabetic mice with *L. casei* CCFM0412 for 12 weeks increased serum levels of GSH, superoxide dismutase (SOD), and glutathione peroxidase (GPx) and reduced ROS and MDA levels [11]. Noteworthy, the results of our study demonstrated that the synbiotic treatment displayed superior antioxidant and anti-inflammatory prowess relative to single therapy. The antioxidant and anti-inflammatory properties of the *L. plantarum* and ASE combination were confirmed in our in vitro study. Our data revealed that *L. plantarum*–fermented ASE (F-ASE) exhibited a considerable reducing ability in the reducing power assay. Furthermore, the results of this study showed that F-ASE had an anti-inflammatory aptitude, evidenced by its ability to suppress nitric acid generation by lipopolysaccharide-stimulated cells in a dose-dependent fashion. Our findings supported other studies reporting the antioxidant and NO production inhibitory activities of *L. plantarum* strains [12, 13]. The observed antioxidant and anti-inflammatory capacities of *L. plantarum*–fermented ASE aligned harmoniously with our in vivo findings wherein the synbiotic treatment markedly amended the oxidative and inflammatory aberrations in the striata and hippocampi of HFD-fed rats. The NO inhibitory potential of the fermented extract was supported by the notably lowered NO striatal and hippocampal levels in the synbiotic-treated HFD-fed group. The anti-inflammatory potential of the *L. plantarum*/ASE combination was further substantiated by the notably curtailed IL-6 levels in the striata and hippocampi of synbiotic-treated rats. In parallel with the in vitro antioxidant capacity of the *L. plantarum*/ASE combination, the synbiotic-treated rats displayed revamped striatal and hippocampal glutathione, MDA, and 8-OHdG levels. In support of the antioxidant proficiency of *Lactobacillus*-based synbiotic therapy, Kleniewska and colleagues [10] showed that co-administration of inulin (400 mg/day) and *L. casei* (4 × 10^8^ CFU/day) for 7 weeks increased serum activities of catalase, SOD, and GPx in healthy subjects.

Prebiotic single therapy in the current study failed to modulate the oxidative status in the striatum, despite significantly stifling the hippocampal oxidative insult. Also, it could neither alleviate the inflammation, amend the energy deficit, nor correct the neurotransmitter imbalance in either of the two brain regions. However, besides amending the hippocampal neurodegenerative aberrations, replenishing the hippocampal BDNF content, and reinstating cognitive function, ASE single therapy also achieved partial modulation of the compromised lipid profile via dampening serum LDL-cholesterol while reinstating serum HDL-cholesterol. Examination of the metabolic profile of *A. officinalis* extract before and after fermentation in the present study revealed the presence of many flavonoid substances, the most important of which are rutin and kaempferol. The observed antioxidant and anti-inflammatory properties of F-ASE could be attributed to the presence of such phenolic compounds. The health-promoting capacity of dietary polyphenol compounds could be a result of their interaction with the gut microbiota, where polyphenols can influence the composition of the microbiota, and beneficial gut bacteria can metabolize polyphenols to produce bioactive compounds with positive health effects [18]. The currently observed memory promoting and neuroprotective attributes of the prebiotic treatment, alone or combined with the probiotic, are comprehensible by virtue of its observed significant ability to relieve the oxidative and neurodegenerative anomalies in the hippocampus. Such prebiotic aptitudes could be explained, at least in part, based on the reported antioxidant and anti-neurodegenerative proficiencies of rutin which can reduce the production of ROS, MDA, and oxidized glutathione levels, increase reduced glutathione levels, and enhance catalase, SOD, and GPx activities [87]. Furthermore, rutin was reported to relieve memory and learning impairments in several rodent models [88, 89], which could be attributed to the capacity of rutin to halt Aβ aggregation and accumulation and plaque generation by decreasing the levels of beta-amyloid precursor protein cleavage enzyme 1 (BACE1) [87]. Additionally, rutin has been reported to enhance BDNF gene expression and reduce tau phosphorylation in rat hippocampi [89, 90].

Accounting for the observed recovery of the striatal and hippocampal neurotransmitter balance in probiotic-treated rats and demonstrating the bi-directional communication in the gut-brain axis, the intestinal microbiota can influence the gut-brain axis via producing neuroactive substances. *Lactobacillus* bacteria have been shown to degrade glutamate and increase GABA levels in the gastrointestinal tract and, as a result, in the central nervous system [91], which could justify their ameliorating effect on learning and memory impairment. In addition, according to pre-clinical reports, commensal bacteria synthesize serotonin, dopamine, norepinephrine, GABA, and acetylcholine as by-products of their metabolic processes [92], which may also play a role in their beneficial effects on cognitive function observed in the present study. Furthermore, *L. plantarum* MTCC 1325 treatment increased acetylcholine levels considerably in the cerebral cortex and the hippocampus [93].

The survival of LAB in the small intestine depends on tolerance to bile salt [94]. In this study, the growth of *L. plantarum* DMS 20174 on an agar plate containing bile salts and CaCl_2_ provided evidence of the existence of BSH activity and its tolerance to bile salts. Our results agree with Hernández-Gómez and co-workers’ findings which revealed a high bile tolerance of *L. plantarum* [95]. The capacity of probiotic strains to lower serum cholesterol levels in hypercholesterolemic patients is usually correlated with their BSH activity [94]. The cholesterol-lowering ability of *L. plantarum* was evident in the current in vivo results, where the HFD-induced elevation in serum cholesterol levels was significantly reverted, restoring the cholesterol levels to normal in the probiotic and synbiotic-treated groups.

The results of this study confirmed that *L. plantarum*, like most of the *Lactobacillus* species, has a “generally regarded as safe” (GRAS) profile [96]; we demonstrated that lactobacilli did not translocate to other internal organs, as indicated by the absence of lactobacilli growth in the spleen, liver, and kidney samples. Furthermore, the results of our in vitro study demonstrated a marked decline in the F-ASE pH after fermentation. This decrease in pH might result from the production of SCFAs by *L. plantarum* DMS 20174 during the fermentation of the ASE. These SCFAs may significantly impact the human gut epithelium [17], possibly contributing to the mechanisms underpinning the neuroprotective attributes of *L. plantarum* therapy.

While the present study provided valuable insights, several limitations must be considered to fully contextualize the findings and guide future research. This study did not explore the full range of bacterial species present in the gut, particularly those of the *Proteobacteria* and *Verrucomicrobia* phyla. Although these species are minor in abundance, they contribute to the diversity of the gut microbiota and play essential roles in interacting with the intestinal mucus layer, influencing both its maintenance and overall gut health. Additionally, the study did not investigate the effects of probiotic and prebiotic consumption on short-chain fatty acid (SCFA) production in the colon, limiting the ability to establish the mechanisms through which daily intake of *Lactobacillus plantarum* DMS 20174 and *Asparagus officinalis* may confer beneficial effects. Moreover, assessing the levels of proteins that maintain intestinal integrity, such as tight junction proteins, would enhance the understanding of whether the observed improvements in cognitive function were linked to enhanced intestinal barrier function. Another important limitation is the use of an animal model for this study, which may not fully replicate human responses. Future research addressing these gaps could provide a more comprehensive understanding of the mechanisms connecting probiotics, prebiotics, and mental health outcomes.

## Conclusion

In conclusion, this investigation supports the detrimental impact of a high-fat diet (HFD) on cognitive and neural function and demonstrates that daily consumption of the *L. plantarum* DMS 20174 probiotic, the ASE prebiotic, or their synbiotic combination provides neuroprotective effects against HFD-induced oxidative stress, neuroinflammation, energy deficits, neurochemical changes, and resulting cognitive impairments. These neuroprotective effects may be mediated through alterations in gut microbiota. Importantly, the synbiotic treatment showed significant improvements in nearly all parameters studied, surpassing the effects of *L. plantarum* or ASE alone, which only partially modulated the endpoints in the hippocampus and striatum. Further research is needed to validate these findings and replicate them in other models of cognitive impairment to determine their potential translation to clinical applications.

## Data Availability

The data that support the findings of this study are available from the corresponding author upon reasonable request.

## Abbreviations

5-HIAA: 5-Hydroxyindoleacetic acid
5-HT: 5-Hydroxytryptamine (serotonin)
8-OHdG: 8-Hydroxy-2′-deoxyguanosine
AChE: Acetylcholinesterase
AD: Alzheimer’s disease
APP: β-Amyloid precursor protein
ASE: *Asparagus officinalis* Extract
ATP: Adenosine triphosphate
Aβ_42_: Amyloid beta 1–42
BSH: Bile salt hydrolase
CFU: Colony-forming unit
DOPAC: 3,4-Dihydroxyphenylacetic acid
F-ASE: Fermented *A. officinalis* extract
F-MRS: Fermented MRS medium
GABA: Gamma-aminobutyric acid
GSH: Reduced glutathione
GSSG: Oxidized glutathione
HDL-C: High-density lipoprotein cholesterol
HFD: High-fat diet
HVA: Homovanillic acid
iNOS: Inducible nitric oxide synthase
LAB: Lactic acid bacteria
LDL-C: Low-density lipoprotein cholesterol
LPS: Lipopolysaccharide
LTP: Long-term potentiation
MDA: Malondialdehyde
MRS: De Man, Rogosa, and Sharpe
MTT: 3-(4,5-Dimethylthiazol-2-yl)-2,5 diphenyltetrazolium bromide
NO: Nitric oxide
p-tau: Phosphorylated tau
ROS: Reactive oxygen species
SCFAs: Short-chain fatty acids
TG: Triglyceride
TC: Total cholesterol
UF-ASE: Unfermented *A. officinalis* extract
